# Golden Gate vectors for efficient gene fusion and gene deletion in diverse filamentous fungi

**DOI:** 10.1007/s00294-020-01143-2

**Published:** 2020-12-24

**Authors:** Tim A. Dahlmann, Dominik Terfehr, Kordula Becker, Ines Teichert

**Affiliations:** Allgemeine und Molekulare Botanik, Ruhr-Universität Bochum, Universitätsstrasse 150, 44801 Bochum, Germany

**Keywords:** Golden Gate cloning, Gene fusion, Gene deletion, Filamentous fungi, Split marker approach, Marker recycling

## Abstract

The cloning of plasmids can be time-consuming or expensive. Yet, cloning is a prerequisite for many standard experiments for the functional analysis of genes, including the generation of deletion mutants and the localization of gene products. Here, we provide Golden Gate vectors for fast and easy cloning of gene fusion as well as gene deletion vectors applicable to diverse fungi. In Golden Gate cloning, restriction and ligation occur simultaneously in a one-pot reaction. Our vector set contains recognition sites for the commonly used type IIS restriction endonuclease *Bsa*I. We generated plasmids for C- as well as N-terminal tagging with GFP, mRFP and 3xFLAG. For gene deletion, we provide five different donor vectors for selection marker cassettes. These include standard cassettes for hygromycin B, nourseothricin and phleomycin resistance genes as well as FLP/*FRT*-based marker recycling cassettes for hygromycin B and nourseothricin resistance genes. To make cloning most feasible, we provide robust protocols, namely (1) an overview of cloning procedures described in this paper, (2) specific Golden Gate reaction protocols and (3) standard primers for cloning and sequencing of plasmids and generation of deletion cassettes by PCR and split-marker PCR. We show that our vector set is applicable for the biotechnologically relevant *Penicillium chrysogenum* and the developmental model system *Sordaria macrospora*. We thus expect these vectors to be beneficial for other fungi as well. Finally, the vectors can easily be adapted to organisms beyond the kingdom fungi.

**Supplementary Information:**

The online version contains supplementary material available at 10.1007/s00294-020-01143-2.

## Introduction

The deletion of genes and the generation of gene fusions are important steps for the functional analysis of genes. With transition into the postgenomic era and the drastic increase in techniques within the field of transcriptomics and proteomics the identification of interesting genes has drastically sped up. However, the initial steps for characterization of a gene, namely the cloning of vectors for expression or localization of a gene product, are still time-consuming or expensive. Here, we describe Golden Gate vector sets for fast and efficient generation of gene deletions and gene fusions primarily intended for fungi, but with modifications applicable to a broader range of organisms.

A number of cloning techniques is available to generate plasmids. Beside the classical restriction and ligation-based cloning, further techniques like yeast recombination or In-Fusion® (Takara Bio) cloning were established enabling faster and—even more important—seamless cloning. For yeast recombination, the baker’s yeast *Saccharomyces cerevisiae* is utilized to merge DNA fragments with homologous regions that can be as short as 30 bp (Hua et al. 1997). With this approach 25 or even more DNA fragments can be combined (Gibson et al. 2008). However, the homologous regions need to be introduced into the DNA fragments used for recombination. This is frequently done by amplifying these fragments using long oligonucleotides that carry the homologous sequences in their 5′ region. However, the length of the oligonucleotides can negatively influence the product yield, even completely suppress amplification. Another disadvantage is that a further cloning step is necessary. The DNA must first be isolated from the yeast before it can be propagated in *Escherichia coli* in a second step. Further, repeats in the cloned fragments can lead to intramolecular recombination and prevent successful cloning (Joska et al. 2014 and our personal observations). In contrast to yeast recombination, the constructs generated with In-Fusion® technology are directly transformed into *E. coli* and therefore one step can be omitted. However, recombination using this technique is most efficient when the homologous regions are at the distal regions of the used DNA fragments. Furthermore, the costs per cloning reaction are higher compared to yeast recombination.

A technique that combines the flexibility of seamless cloning with the versatility of restriction and ligation-based cloning is the Golden Gate assembly. It is based on type IIS restriction enzymes and was described as a cloning platform for construction of vectors for various plants and microorganisms (Engler et al. 2008; Terfrüchte et al. 2014; Agmon et al. 2015; Moore et al. 2016; Hernanz-Koers et al. 2018; Chiasson et al. 2019). Type IIS enzymes are characterized by binding non-palindromic DNA sequences and cutting at a defined distance outside of their binding region. Depending on the enzyme, this trait allows to generate sticky ends with user-defined overhangs of up to four bases such that the original recognition site is no longer present within the DNA fragment after restriction. Thus, the use of type IIS restriction enzymes enables the user to generate seamless constructs through the well-defined ligation of multiple fragments in one reaction. Until now, most Golden Gate-based approaches in fungal molecular genetics had a fixed frame in which for example selected protein tags could only be attached to one terminus or the underlying expression and selection systems served only a narrow host range (Prielhofer et al. 2017; Wu et al. 2018; Nogueira-Lopez et al. 2019).

Here, we present a set of versatile Golden Gate vectors applicable to a wide variety of fungi. The vectors enable C- and N-terminal tagging with EGFP, mRFP and the 3xFLAG-tag using only two PCR fragments generated with three different oligonucleotides. Using this toolbox, a gene of interest (GOI) can be translationally tagged in parallel at different termini with different tags for time- and cost-efficient characterization. Furthermore, constructs for the generation of deletion cassettes using hygromycin B, nourseothricin or phleomycin resistance genes as selection markers are presented. We also generated FLP/*FRT*-based recyclable deletion cassettes containing hygromycin B and nourseothricin resistance markers, enabling further use of these selection markers in subsequent experiments. Lastly, we provide robust protocols for generation of these constructs in combination with DNA fragments of interest to generate vectors ready for transformation experiments. As a proof of principle, we applied these vectors to functional gene analysis and gene deletion in the two filamentous ascomycetes *Penicillium chrysogenum* and *Sordaria macrospora*.

## Materials and methods

### Strains and growth conditions

All filamentous fungal strains used in this study are listed in Table S1. Homologous recombination in yeast was performed in *S. cerevisiae* strain PJ69-4a (James et al. 1996) as previously described (Bloemendal et al. 2012). Restriction-ligation-based cloning as well as propagation of plasmids was performed using standard laboratory protocols (Sambrook and Russel 2001) and *Escherichia coli* XL1 Blue MRF’ (Jerpseth et al. 1992) or JM110 (Yanisch-Perron et al. 1985) as host strains.

### Vector construction

All plasmids used in this study are listed in Table S2, while oligonucleotides used for cloning are listed in Table S3. Oligonucleotides for Golden Gate cloning were either generated with SnapGene® software (from GSL Biotech; available at www.snapgene.com) or using the NEB Golden Gate Assembly Tool (https://goldengate.neb.com/).

Golden Gate vectors in this study are based on vector pEHN8, which contains a fungal expression cassette with the *Aspergillus nidulans gpdA* promoter (Punt et al. 1992), a multiple cloning site (MCS), and the *A. nidulans trpC* terminator [*trpC*(*t*)] (Mullaney et al. 1985). pEHN8 is based on pEHN2 (Pöggeler and Kück 2004), which was cut *Eco*RI, partially cut *Bsp*120I, modified by Klenow fill in, followed by religation of a 6365 bp fragment to generate pEHN5, thereby removing telomeric regions interfering with transformation. Oligonucleotides L-01/L-02 were annealed, cut *Not*I and *Spe*I, and ligated into the corresponding sites of pEHN5 to generate pEHN8.

To exchange the hygromycin B resistance cassette in this vector for the nourseothricin resistance cassette, a 1008 bp *Acc*I fragment from pEHN8 was ligated into a 4903 bp *Acc*I backbone fragment of pEHN1nat (Dreyer et al. 2007), generating pEHN8nat. Since pEHN8nat contains 3 *Bsa*I sites in the *bla* gene, the *trpC* terminator and the vector backbone, these were removed using the Q5 site-directed mutagenesis kit (NEB). In detail, we deleted a 579 bp region in the vector backbone using oligonucleotide pair Mut_pehn8nat_3/Mut_pehn8nat_4, and we further mutated the *Bsa*I sites in *bla* and *trpC*(*t*) using oligonucleotide pairs SM#848/SM#849 and Mut_pehn8nat_1/Mut_pehn8nat_2, respectively, resulting in vector pEHN8nat-GG.

For generation of Golden Gate destination vectors for C-terminal fusion, the *lacZ alpha* fragment and a tag amplificate were hydrolyzed with *Cla*I/*Spe*I and *Spe*I/*Bam*HI, respectively, and ligated into *Cla*I/*Bam*HI-digested pEHN8nat-GG. The 560 bp *lacZ* alpha fragment was amplified from pDrive using oligonucleotides GG-3neu/GG-4. 759 bp and 717 bp PCR fragments for *egfp* and *mRFP* were amplified with oligonucleotides GG-1/GG-2 and GG-C-mRFP-fw/GG-C-mRFP-rv from pIG1783-1 (Pöggeler et al. 2003) and pMSHnat, respectively. pMSHnat was generated by transferring a 681 bp *Nco*I-*Not*I fragment from pMSH, carrying a codon-optimized *mRFP1* gene encoding a Q66T mutation for enhanced fluorescence (Jach et al. 2006; Engh et al. 2010), into the corresponding sites of pRHN1nat. Oligonucleotides FLAG-for1/FLAG-rev1 were annealed to generate the C-terminal 3xFLAG tag with appropriate overhangs.

For generation of Golden Gate destination vectors for N-terminal fusion, the *lacZ α* fragment and a tag amplificate were hydrolyzed with *Cla*I/*Spe*I and *Nhe*I/*Cla*I, respectively, and ligated into *Nhe*I/*Spe*I-hydrolyzed pEHN8nat-GG. The 559 bp *lacZ* alpha fragment was amplified from pDrive using oligonucleotides ClaI_PlacZ_f/LacZ_BsaI_SpeI_r. 751 bp and 709 bp PCR fragments for *egfp* and *mRFP* were amplified with oligonucleotides NheI_EGFP_f/EGFP_GS_BsaI_ClaI_r and GG-N-mRFP-fw/GG-N-mRFP-rv from pIG1783-1 (Pöggeler et al. 2003) and pMSHnat, respectively. Oligonucleotides FLAG-for3/FLAG-rev3 were annealed to generate the N-terminal 3xFLAG tag with appropriate overhangs.

For the cloning of deletion constructs vector, pDest-Amp was generated based on pBluescriptIISK(+) (Stratagene). To remove the naturally occurring *Bsa*I site in the *bla* gene of the initial vector, Q5 site-directed mutagenesis (New England Biolabs) was used in combination with primers SM#848 and SM#849 as described above. Subsequently, a *Bsa*I-flanked integration site was introduced by ligation of *Eco*RI hydrolyzed mutated vector with annealed oligonucleotides GG_Int1 and GG_Int2.

For convenient cloning, vectors harboring resistance cassettes flanked by appropriate *Bsa*I recognition sites were generated. For generation of standard selection cassette donor vectors, resistance cassettes containing the *trpC* promoter and a resistance gene were PCR amplified and cloned into pDrive (*hph*, *phleo*) or pJet1.2 (*nat1*) using the Qiagen PCR Cloning Kit (Qiagen) or the CloneJET PCR cloning kit (Thermo Fisher Scientific). For pGG-phleo, pGG-hph and pGG-nat1, the resistance cassette was amplified using primer pair GG_phleo_fw/GG_phleo_rv, primer pair GG-KO-hph-fw/GG-KO-hph-rv and primer pair GG-nat-fw/GG-nat-rv from pD-Phleo (Hoff et al. 2010a), pDrivehph (Nowrousian and Cebula 2005) and pEHN1nat (Dreyer et al. 2007), respectively.

To construct vectors for using the FLP/*FRT* marker recycling system, two *Bsa*I recognition sites within the flippase gene of pFlip (Bloemendal et al. 2014) had to be removed. This was done in two consecutive steps using the Q5 site-directed mutagenesis kit (NEB) together with primer pairs pcflp_mut1_fw/pcflp_mut1_rv and pcflp_mut2_fw/pcflp_mut2_rv, generating pFlip-mut2. For generation of pGG-Flip-hph, two *Bsa*I sites were introduced into pFlip-mut2 by oligonucleotide annealing and restriction/ligation. Specifically, oligonucleotides Bsa-L1-fw and Bsa-L1-rv were annealed and introduced via* Pst*I and *Eco105*I, yielding pFlip-mut2-Bsa-Linker1, and oligonucleotides Bsa-L2-fw and Bsa-L2-rv were annealed and introduced via* Hin*dIII and *Bgl*II, yielding pGG-Flip-hph. For generation of pGG-Flip-nat1, *nat1* was amplified from pEHN1nat (Dreyer et al. 2007) using primer pair nat-Kass-fw/nat-Kass-rv and transformed into yeast together with *Aat*II-digested pRS-Kassette (Bloemendal et al. 2014), yielding pRS-nat-Kassette. Subsequently, an 1872 bp *Bam*HI-*Nco*I fragment from pRS-nat-Kassette was cloned into the *Bam*HI and *Nco*I sites of pGG-Flip-hph, generating pGG-Flip-nat1.

### Golden Gate reaction

Reactions were set up in 0.5-ml PCR tubes according to Terfrüchte et al. (2014) with minor modifications. In detail, 1 µl T4 DNA ligase (NEB; 1 U/µl) and 1 µl *Bsa*I or *Bsa*I-HFv2 (NEB; 20 U/µl) were mixed in 15 µl reactions in 1 × T4 DNA ligase buffer (NEB). For gene fusion, equal amounts (40–80 fmol each) of the respective destination vector and the purified, precipitated or sub-cloned PCR product for the GOI were used. For gene deletion vectors, amplified and purified 5′ and 3′ flanks of the target region, pDest-Amp and a cassette donor vector were mixed in a 2:2:2:1 ratio (see Table S6). Golden Gate reactions were performed in a PCR cycler as described in Terfrüchte et al. (2014) with slight modifications (Table S4): After an incubation at 37 °C for 30 (protocol I) or 90 min (protocol II) for generation of gene fusion or deletion vectors, respectively, 50 cycles of 37 °C 2 min, 16 °C 5 min were performed, followed by 5 min 37 °C, 5 min 50 °C and 5 min 80 °C. When cloning fragments with internal *Bsa*I sites, the 50 cycles of 37 °C 2 min, 16 °C 5 min were directly followed by 5 min at 80 °C (protocol III).

Golden Gate reactions were transformed into *E. coli* XL1-Blue MRF and white colonies chosen for plasmid preparation. Plasmids were subjected to restriction analysis, and at least one positive sample for each construct was analyzed by Sanger sequencing to prove seamless cloning and correct insert sequence.

### Generation of transgenic fungal strains

Transformation of *S. macrospora* was performed as described, but without caylase (Dirschnabel et al. 2014). Transformants were inoculated on biomalt corn medium (BMM) using the appropriate antibiotics [nourseothricin (50 µg/ml) or hygromycin B (80 U/ml)]. Genomic DNA was isolated by phenol/chloroform extraction from cultures grown in liquid BMM.

To generate *S. macrospora* Δidc3 (SMAC_00214), deletion vector pKO-idc3 was generated by homologous recombination in yeast as described (Colot et al. 2006; Bloemendal et al. 2012). Specifically, the *hph* resistance cassette was cut from pDrivehph (Nowrousian and Cebula 2005) with *Eco*RI and transformed in yeast strain PJ69-4a (James et al. 1996) together with *Eco*RI/*Xho*I-digested pRS426 (Christianson et al. 1992) and 5′- and 3′-flanking regions amplified from genomic DNA with primer pairs 214-5fw/214-5rv and 214-3fw/214-3rv, respectively. A 3404 bp *Bam*HI fragment from pKO-idc3 was transformed into *S. macrospora* Δku70 (Pöggeler and Kück 2006). For generation of *S. macrospora* Δefd16, the deletion cassette was amplified from pKO-efd16 using primer pair 2986-5fw/2986-3rv and transformed into *S. macrospora* Δku70. Hygromycin B-resistant transformants were crossed to spore color mutant fus (Nowrousian et al. 2012) to generate homokaryotic isolates devoid of the Δku70 background. Deletion of the *idc3* and *efd16* genes was verified by PCR analysis (Supplementary Figures S1 and S2).

For generation of deletion strain Δtih, a non-homologous end joining (NHEJ)-deficient and hygromycin B-resistant recipient strain (Herzog and Pöggeler, unpublished results) was transformed with two PCR fragments amplified from pKO-tih with primer pairs tih-KO-5fw/nat_split_rev_new and nat-split-fw_new/tih-KO-3rv (Supplementary Tables S5 and S6). Hygromycin B- and nourseothricin-resistant transformants with red ascospores were isolated, analyzed by PCR for correct integration of the deletion cassette, and crossed to wildtype to dispose of the NHEJ-deficiency and to recycle the *nat1* marker. Red-spored isolates showing hygromycin B as well as nourseothricin sensitivity were analyzed by PCR to verify marker recycling (Supplementary Figure S3).

Transformation of *P. chrysogenum* was performed as described by Bull et al. (1988) with modifications as follows: Flasks containing complete culture medium (CCM) were inoculated with 1 × 10^7^ spores and cultures were incubated as described in Wolfers et al. (2015). Fungal protoplasts were generated by incubation of the harvested mycelium with VinoTaste® Pro (Novozymes) (solution of 1 g VinoTaste® Pro in 30 ml 0.9 M NaCl) for 2 h at 27 °C and 100 rpm. After DNA transformation and incubation at 27 °C for 24 h on CCMS (CCM with 20% sucrose), protoplasts were overlaid for selection with 11 ml 0.8 M NaCl containing 0.8% agar and 200 µg/ml nourseothricin or 40 µg/ml phleomycin, respectively. Transformants were transferred onto selective CCM plates after a further 3–4 days. Validation of the successful deletion of the targeted DNA region was performed by Southern blotting and PCR analysis (Supplementary Figures S4 and S5).

### Phenotypic analysis

For validating fruiting body formation, *S. macrospora* strains were grown on BMM for seven days and imaged with a STEMI 2000-C stereomicroscope (Zeiss) equipped with an AxioCamERc5s (Zeiss) using ZEN software (Zeiss). Ascospore formation was assayed after ten days of growth on BMM using an Axiophot microscope (Zeiss) equipped with an AxioCam camera (Zeiss) using ZEN software (Zeiss). Images were processed with Adobe Creative Suite 6 (Adobe Corp.).

### Fluorescence microscopy

*S. macrospora* was inoculated on BMM-coated microscope slides and incubated for 2 days in a Petri dish as described (Engh et al. 2007b). *P. chrysogenum* conidiospores were inoculated on a CCM-coated microscopic slide and incubated for 2 days at 27 °C. Fluorescence and differential interference contrast (DIC) microscopy were carried out with an AxioImager.M1 microscope (Zeiss) equipped with a Photometrix Cool SnapHQ camera (Roper Scientific) and a SpectraX LED lamp (Lumencor) using MetaMorph software (Universal Imaging). GFP and mRFP fluorescence were detected using filter set 41017 (Chroma Technology; HQ470/40, HQ525/50, Q495lp) and 49008 (Chroma Technology; excitation filter HQ560/40, emission filter ET630/75 m, beam splitter T585lp), respectively. Images were processed with MetaMorph, ImageJ (Schneider et al. 2012) and Adobe Creative Suite 6 (Adobe Corp.).

### Protein purification

Proteins were extracted from *S. macrospora* strains grown for 4–5 days in BMM liquid culture. Dried mycelium was ground in liquid nitrogen and suspended in extraction buffer (100 mM Tris–HCl pH 7.6, 250 mM NaCl, 10% glycerol, 0.5% NP-40, 2 mM EDTA, 2 mM DTT, 1% SDS, protease inhibitor cocktail IV (1:100, Calbiochem), 1 mM PMSF, 1 mM benzamidine). After centrifugation at 11,000 rpm for 30 min, the supernatant was subjected to SDS-PAGE and Western blotting. GFP-tagged IDC3 was detected with anti-GFP antibody (Living Colors JL-8; TaKaRa Bio Europe/Clontech, Saint-Germain-en-Laye, France) and an anti-mouse IgG horseradish peroxidase-linked secondary antibody as described before (Nordzieke et al. 2015).

## Results and discussion

### Generation of Golden Gate destination vectors for gene fusions

Our first aim was to establish Golden Gate plasmids for fast and easy generation of gene fusions for complementation of deletion strains and functional analysis of genes. We intended the plasmids to be convenient for a number of different fungi, including *Acremonium chrysogenum*, *P. chrysogenum* and *S. macrospora*, three fungi frequently used in our studies (Bloemendal et al. 2014; Teichert et al. 2014, 2020; Dahlmann et al. 2015; Terfehr et al. 2017). We chose three different tags: the two fluorescent proteins GFP and monomeric red fluorescent protein (mRFP) and the 3 × FLAG affinity purification tag. GFP has been used in numerous fungi, including our three main study objects *A. chrysogenum*, *P. chrysogenum* and *S. macrospora*, in translational fusions to localize proteins as well as for organelle labeling (Pöggeler et al. 2003; Hoff et al. 2005; Engh et al. 2007b; Veiga et al. 2012; Fernández-Aguado et al. 2013; Bloemendal et al. 2014; Nordzieke et al. 2015; Wang et al. 2018; Werner et al. 2019). Red fluorescent proteins (RFPs) are often used to label a second protein or an organelle for co-localization with a GFP-labeled protein. For our Golden Gate plasmids, we chose a previously described monomeric RFP (mRFP) version codon-optimized for *S. macrospora* (Engh et al. 2010). Localization studies with mRFP have already been performed to co-localize transcription factors with nuclear markers in *A. chrysogenum*, and a histone 2A-mRFP fusion protein has been applied as nuclear marker in *S. macrospora* (Hu et al. 2015; Wang et al. 2018; Lütkenhaus et al. 2019; Schmidt et al. 2020). GFP fusions can also be used for affinity purification and subsequent mass spectrometry, or for chromatin immunoprecipitation (ChIP)-seq, as has been described for *P. chrysogenum* and *S. macrospora* (Becker et al. 2015; Nordzieke et al. 2015; Steffens et al. 2016; Werner et al. 2016). Both GFP and mRFP are also known to be suitable in a wide variety of different filamentous fungi (Lorang et al. 2001; Pöggeler et al. 2003; Toews et al. 2004; Steinberg and Perez-Martin 2008). The 3xFLAG-tag was also included because of its small size (3 kDa) and its application in established protein purification protocols for filamentous fungi (Honda and Selker 2009; Bloemendal et al. 2014; Nordzieke et al. 2015).

Proteins may contain targeting sequences or important domains at their N- or C-termini that could be functionally blocked by fusion of a tag. We therefore generated Golden Gate plasmids for N- and C-terminal tagging with GFP, mRFP and 3xFLAG. In any case, tag fusion can cause misfolding of the target protein (Booher and Kaiser 2008; Sung et al. 2008), and this has been described to be circumvented by inserting a glycine-serine (GS) linker between target protein and tag (e.g., Honda and Selker 2009). Therefore, our Golden Gate plasmids contain a GSGSG linker coding sequence between tag and GOI. For expression of the fusion constructs, we chose a constitutive expression system consisting of the *Aspergillus nidulans gpdA* promoter and *trpC* terminator that has been used in numerous fungi (Fowler and Berka 1991; Mei et al. 2019).

**Fig. 1:**
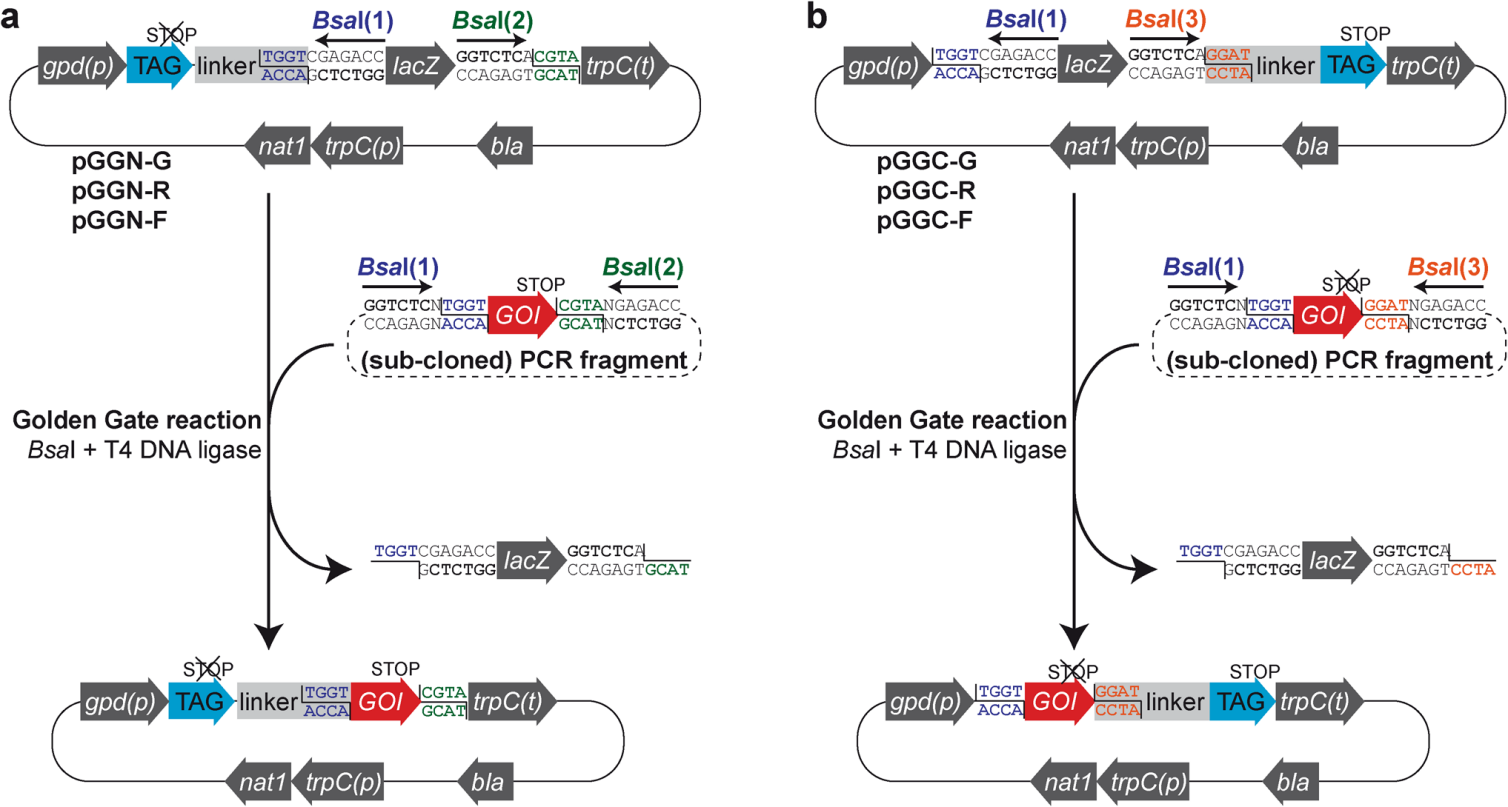
Workflow describing the generation of gene fusions. **a** For N-terminal tagging, the gene of interest (GOI) is amplified with oligonucleotides containing *Bsa*I sites *Bsa*I(1) and *Bsa*I(2), generating overhangs 1 (blue) and 2 (green). Subsequently, these (sub-cloned) PCR fragments are used in a Golden Gate reaction with Golden Gate fusion vectors pGGN-G, pGGN-R, or pGGN-F, to generate GFP-, mRFP- or FLAG-fusions, respectively. **b** For C-terminal tagging, the GOI is amplified without stop codon with oligonucleotides containing *Bsa*I sites *Bsa*I(1) and *Bsa*I(3), generating overhangs 1 (blue) and 3 (orange). Subsequently, these (sub-cloned) PCR fragments are used in a Golden Gate reaction with Golden Gate fusion vectors pGGC-G, pGGC-R or pGGC-F, to generate GFP-, mRFP- or FLAG-fusions, respectively. Golden Gate reaction protocols are given in Table S4, while standard oligonucleotides for cloning and Golden Gate reaction setups are given in Tables S5 and S6, respectively

The Golden Gate system relies on type IIS restriction enzymes that bind non-palindromic DNA sequences and cut at a defined distance outside of their binding region. We first analyzed the frequency of recognition sites for type IIS restriction enzymes *Bbs*I, *Bsa*I and *Bsm*BI in the genome sequences of *A. chrysogenum*, *P. chrysogenum* and *S. macrospora* (Nowrousian et al. 2010; Teichert et al. 2012; Specht et al. 2014; Terfehr et al. 2014). We observed no significant differences in the number of sites or the distribution of sites within annotated features and intergenic regions between the analyzed enzymes (supplementary Figure S6). *Bsa*I is commercially available as a Golden Gate-optimized version and readily performs endonucleolytic cleavage in ligation buffer, making it a very convenient choice for our system. We designed our Golden Gate plasmids to have different overhangs for the cloning of N-terminal fusions [*Bsa*I(1), *Bsa*I(2), Fig. 1a] and C-terminal fusions [*Bsa*I(1), *Bsa*I(3), Fig. 1b] (see also Tables S2, S5).

To allow for easy selection of positive clones, the *lacZ* alpha fragment was inserted between both *Bsa*I sites to enable blue-white screening. The *lacZ* gene is completely removed from the vector in a successful Golden Gate ligation, rendering the selection system very strong (Fig. 1). As a fungal selection marker, we chose the *nat1* gene conferring nourseothricin resistance, because it is widely applicable in fungal systems (Kück and Hoff 2006; Hoff et al. 2010b; Mehrabi et al. 2015; He et al. 2020).

Figure 1 gives an overview of our gene fusion Golden Gate plasmids and the workflow to generate target plasmids. In addition, supplementary Tables S5 and S6 list all primers and elements required to generate fusion tag vectors or deletion vectors with Golden Gate plasmids from this study. For example, to generate an N-terminal GFP fusion construct, the GOI including its intrinsic stop codon has to be amplified using primers GG-C/N-fw/GG-N-rv (Table S5). The purified product is then mixed with destination vector pGGN-G in a Golden Gate reaction according to supplementary Table S6 with *Bsa*I and ligase using a Golden Gate protocol I (Table S4). For C-terminal GFP fusion, the GOI without stop codon has to be amplified using primers GG-C/N-fw/GG-C-rv (Tables S5, S6). The purified product is then mixed with destination vector pGGC-G in a Golden Gate reaction. Certainly, primers can also be designed using other means, e.g., the NEB Golden Gate Assembly Tool (https://goldengate.neb.com/). However, we provide primer sequences with standard 5′ extensions for cloning, where only the GOI-specific 17–23 nucleotides have to be added (Table S5). These primers contain the *Bsa*I binding site *ggtctc*, additional 5′ nucleotides necessary for *Bsa*I binding to DNA as well as the overhang sequences compatible to the destination vectors generated in this study.

### Application of the Golden Gate system for functional analysis of developmental genes in *S. macrospora*

**Fig. 2:**
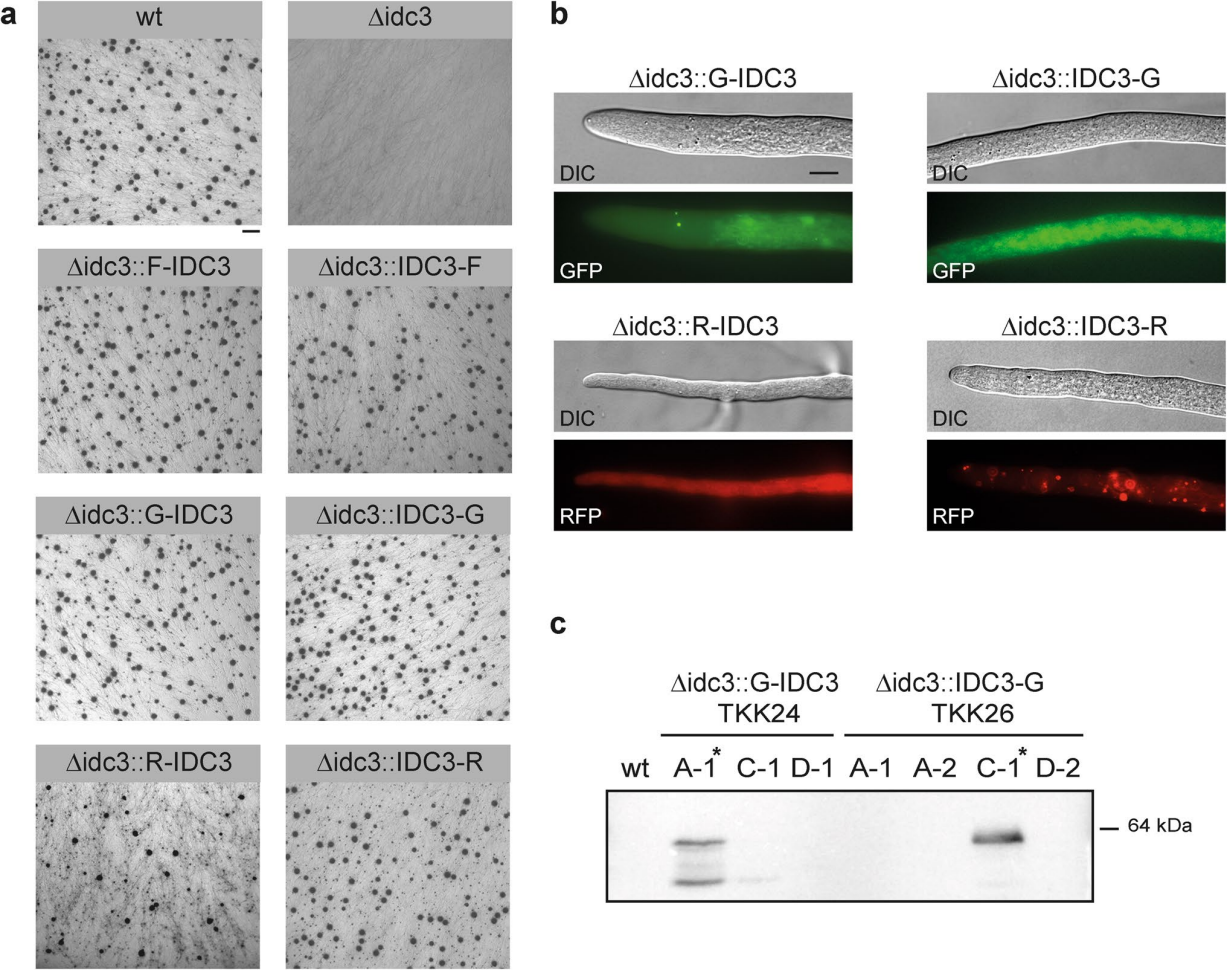
Functional analysis of IDC3 in *S. macrospora*. **a** Phenotypic analysis of Δidc3 and complemented strains. Strains were grown for 7–10 days on BMM. Note that the complemented strains form perithecia like wild type (black dots). Bar, 1 cm. **b** Localization of IDC3. GFP- and mRFP-tagged IDC3 localizes to vesicular structures. DIC: Differential interference contrast microscopy. Bar, 10 µm. **c** Western analysis of strains expressing GFP-tagged IDC3 with anti-GFP antibodies. Strains carrying constructs for N-terminal GFP-fusions (TKK24) as well as C-terminal GFP-fusions (TKK26) were analyzed. Two strains show the expected signal of the GFP-IDC3 fusion protein at a size of 64 kDa. Strains TKK24A-1 and TKK26C-1 used in further analyses are marked by asterisks

As a candidate gene for functional analysis in *S. macrospora*, we chose *SMAC_00214*. This gene was previously identified as a target gene of zinc finger transcription factor PRO1 in RNA-seq and ChIP-seq analysis, and *N. crassa* strains lacking the homologous gene *NCU00938* are sterile in homozygous crosses as well as when used as the female partner in crosses to *N. crassa* wild type (Steffens et al. 2016). *SMAC_00214* is homologous to *Epichloë festucae symC* and *Podospora anserina idc3* (Green et al. 2017; Lalucque et al. 2017) and was thus renamed *idc3*. Deletion of *S. macrospora idc3* resulted in a sterile phenotype, as described for other ascomycetes (Steffens et al. 2016; Green et al. 2017; Lalucque et al. 2017) (Fig. 2a).

For mutant complementation and localization of IDC3 in *S. macrospora*, we generated N- and C-terminal fusions of IDC3 to GFP, mRFP and 3xFLAG, using the above-described Golden Gate vectors. Table 1 shows the corresponding cloning efficiencies. For each plasmid, one Golden Gate reaction was performed and transformed into *E. coli*, yielding varying numbers of colonies and a different percentage of white colonies. However, from three randomly chosen white colonies per plasmid, at least one yielded the desired fusion tag construct. Transformation of Δidc3 with each of the six vectors resulted in fertile strains, indicating that the mutant defect was indeed caused by lack of *idc3* and that the gene fusions were functional (Fig. 2a). It should be noted, however, that only 10% of transformants carrying pGGN-R-IDC3 regained fertility, indicating that this construct is less commendable to work with.

**Table 1:** Efficiency of generation of Golden Gate vectors for *idc3*

Plasmid	pGGN-G-IDC3	pGGN-R- IDC3	pGGN-F- IDC3	pGGC-G- IDC3	pGGC-R- IDC3	pGGC-F- IDC3
# Colonies	111	154	135	26	169	Incalculable
# White colonies	94	108	38	15	154	Incalculable (18 blue colonies)
% White colonies	85	70	28	58	91	nd^a^
# Minipreps	3	3	3	3	3	3
# Positive clones	2	1	2	3	3	2

^a^Not determined

Fusion to diverse fluorescent tags at different positions might give mixed results, which was one of the reasons why we generated vectors for N- as well as C-terminal tagging with different proteins. Previously, C-terminally RFP-tagged SymC was described to localize to vacuoles and vesicles in *E. festucae* (Green et al. 2017). For *S. macrospora*, microscopic analysis showed localization of C-terminally, but not N-terminally mRFP-tagged IDC3 to vesicular structures (Fig. 2b). GFP-tagged IDC3 showed rare localization to punctate structures and a net-like localization reminiscent of tubular vacuoles. These results indicate interference of the fused fluorescent protein as well as its position on IDC3 localization, highlighting the importance of generating different fusions, which is quite feasible with the vector set generated in this study.

GFP-fusion strains were further subjected to protein extraction, and Western blot analysis using anti-GFP antibodies showed the presence of the fusion proteins in transformants TKK24A-1 and TKK26C-1 (Fig. 2c). These strains now open up new research avenues, since they can be used for pulldown experiments to identify IDC3 interaction partners. Such interaction partners have not been described yet in any system and could give insight into the function of the protein, which remains elusive.

### Application of the Golden Gate system for protein localization of MAT1-1-1 in *P. chrysogenum*

**Fig. 3:**
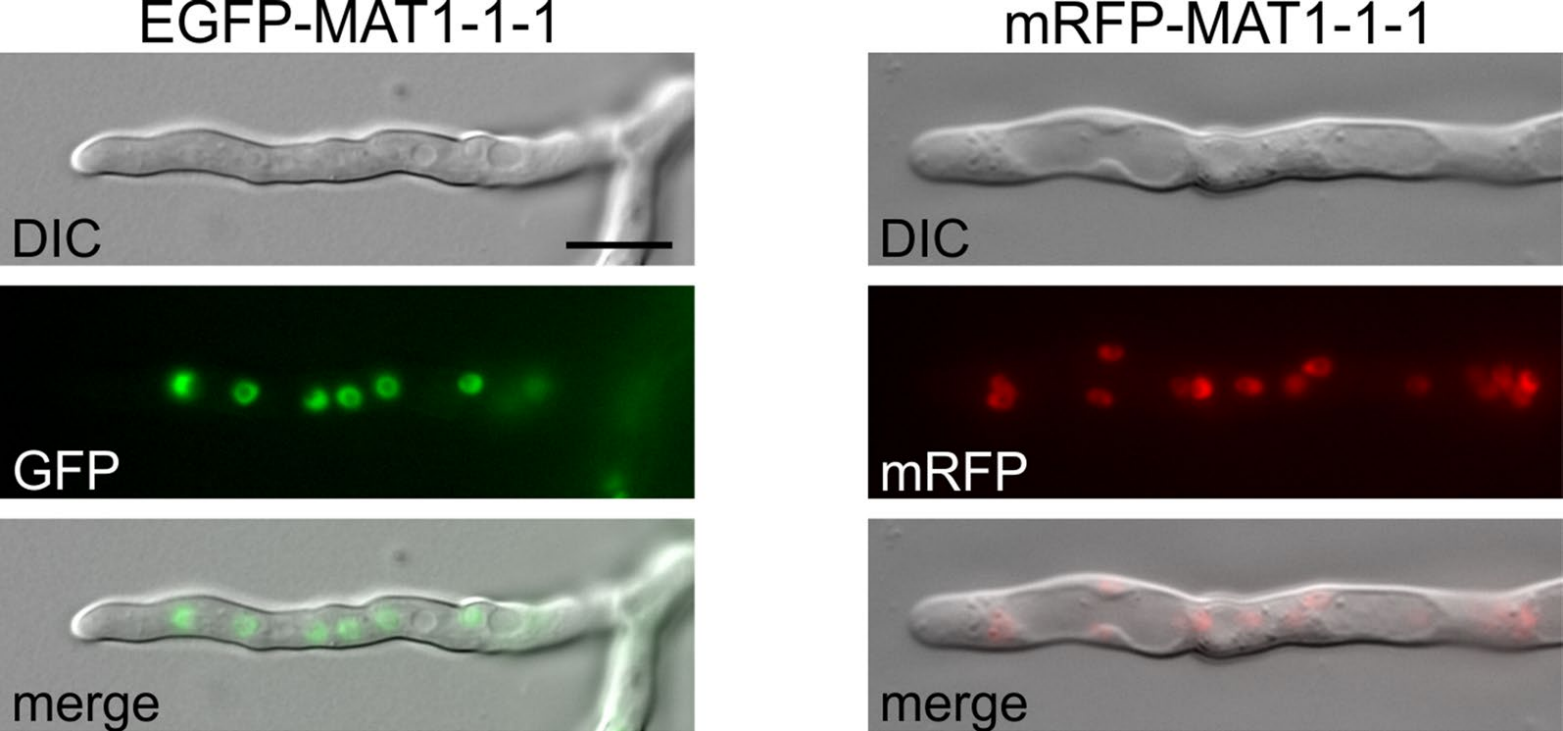
Localization of MAT1-1-1 in *P. chrysogenum*. MAT1-1-1 fused N-terminally to EGFP or mRFP localizes to the nucleus. DIC, differential interference contrast microscopy; bar, 10 µm

To prove the applicability of the Golden Gate fusion vectors for a variety of fungi, we generated vectors for localization of *P. chrysogenum* MAT-1-1-1. This mating type-specific transcription factor regulates pellet formation, branching of conidial germ tubes and the expression of target genes important for sexual development as well as other functions (Böhm et al. 2013; Becker et al. 2015). An 1113 bp *mat-1-1-1* amplicon was generated using oligonucleotides GGN-PcMAT1-rev/GG-PcMAT1-for from pGFP-MAT1 (Becker et al. 2015). Since C-terminal tagging of MAT1-1-1 yields non-functional fusion proteins, we set up two Golden Gate reactions with the *mat1-1-1* amplicon and pGGN-G and pGGN-R, for N-terminal tagging, according to Table S6 using Golden Gate protocol I (Table S4). Transformation of pGGN-G-Pcmat1 and pGGN-R-Pcmat1 vectors into *P. chrysogenum* Δmat1 yielded strains with a deviant pellet formation, as described for *MAT1-1-1* overexpression strains (Böhm et al. 2013). N-terminal tagging with GFP as well as mRFP showed nuclear localization of MAT1-1-1 (Fig. 3), as previously reported. Thus, the codon-optimized *mRFP* version is also functional in *P. chrysogenum*. Furthermore, the EGFP tag used in our Golden Gate vectors is also well suited for ChIP-seq experiments, as demonstrated for N- and C-terminal GFP fusion of MAT1-1-1 and PcVelA, respectively (Becker et al. 2015, 2016). Taken together, our gene fusion vector set is applicable for characterization of gene functions in diverse ascomycetes.

#### Generation of Golden Gate vectors for gene deletion

Generation of gene deletions is of major importance to elucidate the function of a gene. We previously generated deletion vectors for diverse fungi by conventional restriction/ligation, InFusion and yeast recombination cloning (Engh et al. 2007b; Bloemendal et al. 2012, 2014). However, all these systems have drawbacks. In restriction/ligation, two consecutive reactions are performed to integrate 5′ and 3′ flanking regions, InFusion cloning is rather expensive, and yeast recombination is time-consuming and does not allow for repetitive sequences. This is of special importance when using previously described FLP/*FRT* deletion cassettes for marker recycling, which need repetitive *FRT* sites for site-specific recombination of the deletion cassette (Kopke et al. 2010; Bloemendal et al. 2012, 2014; Teichert et al. 2017).

**Fig. 4:**
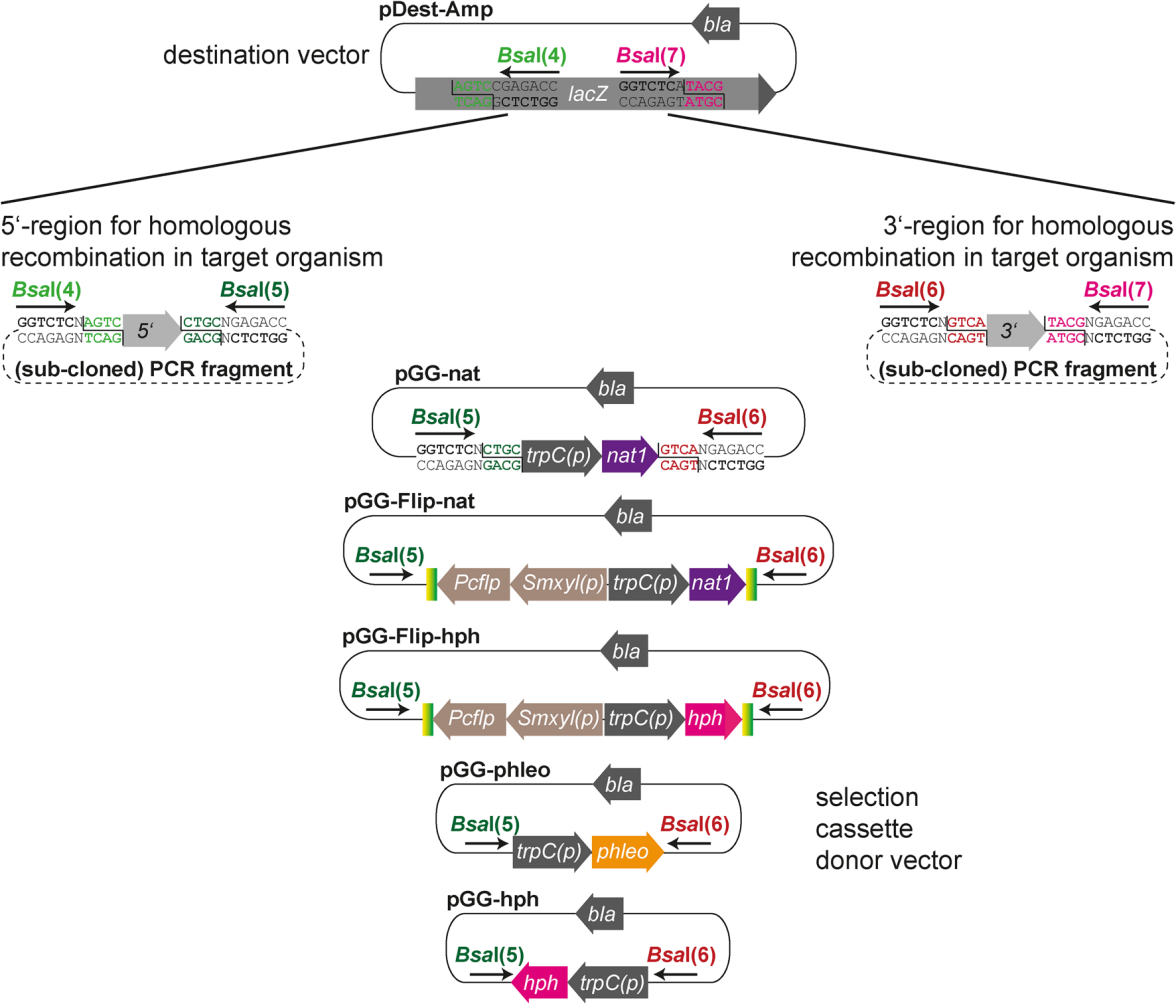
Generation of deletion vectors using the Golden Gate system. Each reaction contains pDest-Amp, a selection marker cassette suitable for the target organism from a selection cassette donor vector, and two PCR fragments for homologous recombination in the target organism. These fragments have to be amplified with oligonucleotides that contain *Bsa*I sites generating the required overhangs 4 (light green) and 5 (dark green) for the 5′ flanking region and overhangs 6 (red) and 7 (pink) for the 3′ flanking region, respectively. Reactions are set up according to Table S6 and using Golden Gate protocol II (Table S4). Standard primers for cloning and sequencing are given in Table S5

To make the deletion vector set applicable for diverse systems, we generated a pDest-Amp vector with a mutation in the *Bsa*I site of the *bla* gene and generated different donor vectors for antibiotic marker cassettes, flanked by *Bsa*I sites (Fig. 4, Tables S2, S6). For standard gene deletion vectors, which lead to strains with the resistance cassette remaining in the mutant genome, we generated donor vectors with the phleomycin, the hygromycin B and the nourseothricin resistance cassette. For FLP/*FRT*-mediated marker recycling, we generated donor vectors with the hygromycin B and the nourseothricin resistance cassette, based on pFlip (Bloemendal et al. 2014), eliminating two *Bsa*I sites from the *Pcflp* gene (see “Materials and methods”). An overview of the deletion vector modules is given in Fig. 4. pDest-Amp carries *Bsa*I sites *Bsa*I(4) and *Bsa*I(7) within the *lacZ* α fragment, while the selection marker cassettes are bordered by *Bsa*I sites *Bsa*I(5) and *Bsa*I(6), each generating specific overhangs. Thus, the 5′ and 3′ flanking region has to be amplified with primers containing *Bsa*I(4) as well as *Bsa*I(5) sites, and *Bsa*I(6) as well as *Bsa*I(7) sites, respectively. These primers can be generated either manually, using cloning software or the NEB Golden Gate Assembly Tool (https://goldengate.neb.com/). However, for fast and easy construction, we provide sequence information for standard primers GG-5fw/GG5-rv and GG-3fw/GG3-rv for the amplification of 5′ and 3′ flanking regions, respectively, in Table S5. In these primers, only the case-specific 17–23 nucleotides have to be added at the 3′ end. Reactions are then set up as specified in Table S6 for either the standard or the marker recycling deletion vectors. Importantly, for generation of deletion vectors, we used a different Golden Gate protocol (protocol II in Table S4): The selection cassette donor vector was used in less quantity, and the Golden Gate reaction was started with a 90 min’ incubation at 37 °C, to ensure complete digestion of the cassette donor vector. This incubation can be prolonged if Golden Gate reactions result in recovering just the selection cassette donor vector.

To test the applicability of the deletion vector system, we generated deletion vectors for two *S. macrospora* and two *P. chrysogenum* genes with different selection marker cassettes according to the protocols, primers and reactions specified in supplementary Tables S4–S6. For *S. macrospora*, we chose *edited in fruiting body development* (*efd*) 16 (*SMAC_02986*), a gene identified in a previous proteogenomics approach to undergo stop loss A-to-I RNA editing at the transcript level (Blank-Landeshammer et al. 2019), and *tih* (*SMAC_05650*), a gene encoding trihydroxynaphthalene reductase from the melanin biosynthesis pathway (Engh et al. 2007a). A point mutation in a splice site of the *tih* gene was previously shown to cause the red-spored phenotype of the *S. macrospora* fus mutant (Nowrousian et al. 2012).

To generate pKO-efd16, a Golden Gate reaction was performed according to Fig. 4 and Table S6 with pDest-Amp, pGG-hph and two PCR fragments corresponding to 1 kb each 5′ and 3′ flanking regions amplified from *S. macrospora* genomic DNA with primer pairs 2986-5fw/2986-5rv and 2986-3fw/2986-3rv, respectively. The deletion cassette was amplified from pKO-efd16 using primers 2986-5fw/2986-3rv and transformed into *S. macrospora* Δku70. Two primary transformants were crossed to the fus mutant, and deletion of the *efd16* gene was verified by PCR in three ascospore isolates TIT8B-1S1, -1S2 and -1S3 (Supplementary Figure S2). The phenotype of the mutant will be described elsewhere.

**Fig. 5:**
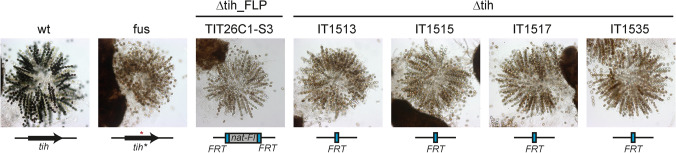
Deletion of the *tih* gene using a Golden Gate-cloned *nat1* marker recycling cassette results in the expected brown-spored phenotype. Strains were grown for 10 days on BMM plates and perithecia were cracked to analyze ascus rosettes. Note that wild type (wt) generates asci with black ascospores, while Δtih strains generate asci with brown spores, like the fus mutant that carries a mutated *tih* gene (tih*) with a single base pair mutation (red asterisk). Transformant TIT26C1-S3 still carries the nourseothricin resistance gene within the *nat*-flipper cassette (nat-Fl), while ascospore isolates IT1513, IT1515, IT1517 and IT1535 are marker-free strains

The *tih* deletion vector pKO-tih was generated according to Fig. 4 with Golden Gate protocol II and using pGG-Flip-nat1 as a selection cassette donor vector. The flanking regions of *tih* were amplified using primer pairs tih-KO-5fw/tih-KO-5rv and tih-KO-3fw/tih-KO-3rv. For fungal transformation, we employed the split marker system and generated two PCR fragments with an overlap in the *nat1* gene. Four fertile primary transformants showed a mixture of red and black ascospores, indicating successful deletion of the *tih* gene. A red ascospore was isolated from transformant TIT26C-1S3 and the strain subsequently crossed to wild type. Red ascospore progeny indicative of a Δtih genotype was analyzed by PCR and microscopy (Fig. 5, Fig. S3). As shown before, marker recycling occurs during the crossing process (Teichert et al. 2017); thus, the ascospore isolates IT1513, IT1515, IT1517 and IT1535 are nourseothricin-sensitive *tih* deletion strains.

To demonstrate the applicability of the pGG-nat1 deletion cassette in *P. chrysogenum*, two genes were selected for gene deletion using the *Pcku70*-deficient recipient strain Δku70FRT2 (Kopke et al. 2010). Genes *EN45_082340* and *EN45_100120* were selected, because both show a significantly upregulated gene expression in the formerly industrially used penicillin producer strain P2niaD18 compared to the wild-type strain NRRL 1951 (Terfehr et al. 2017). *EN45_082340* encodes a 3-hydroxyisobutyrate dehydrogenase, which is putatively involved in the degradation of the penicillin precursor amino acid valine, and *EN45_100120* encodes a putative lipoamide acyltransferase, which is predicted to be part of the branched-chain α-keto acid dehydrogenase complex. For the construction of the deletion cassettes, 5′- and 3′-flanking regions of around 1 kb each were amplified by PCR. For *EN45_082340* the primer pairs 082340-5fw/082340-5rv and 082340-3fw/082340-3rv and for *EN45_100120* the primer pairs 100120-5fw/100120-5rv and 100120-3fw/100120-3rv were used. The Golden Gate reaction was performed with the amplicons, pDest-Amp and pGG-nat1 as described in Fig. 4 and Table S6 to generate pKO-082340 and pKO-100120, respectively.

A special factor should be noted in the context of cloning the pKO-082340 deletion vector. The 3′-flank of gene *EN45_082340* contains an internal *Bsa*I recognition site, which is located at position 473 of the 3′-flank and 433 bp in front of the start codon of the following gene. Thus, the alternative Golden Gate cloning protocol III for internal *Bsa*I sites was used (Table S4). In principle, the presence of a single additional *Bsa*I restriction sites can be ignored if the generated overhang is not compatible with the overhangs generated by *Bsa*I digestion in the destination or resistance cassette vectors [here, the pre-designed sites *Bsa*I(4), (5), (6), (7)] and if the two final restriction steps are skipped according to protocol III in Table S4. Although the cloning efficiency is decreased by the presence of additional *Bsa*I sites, it is usually possible to produce sufficient amounts of correctly ligated DNA products, as demonstrated for pKO-082340. The deletion cassettes were amplified from pKO-082340 and pKO-100120 using primers 082340-5fw/082340-3rv and 100120-5fw/100120-3rv, respectively. Deletion of both genes in a *Pcku70*-deficient P2niaD18 strain (∆ku70FRT2) was performed as described previously (Hoff et al. 2010b) and verified by Southern blotting and PCR (Supplementary Figures S4, S5). The phenotype of the deletion strains will be discussed elsewhere.

As an alternative to the above-described strategy for cloning of fragments with multiple *Bsa*I sites, the Golden Gate cloning strategy is adaptable to introduce a single-point mutation in each *Bsa*I recognition site by PCR. The region of interest (ROI) is divided into fragments according to the present *Bsa*I binding sites. Thus, for each internal *Bsa*I site, two additional primers must be designed. One primer is a standard 18–20 nt oligomer that maintains the original *Bsa*I recognition site, which must be oriented in 5′-to-3′ direction in the primer, thus facing into the PCR fragment to be generated. During Golden Gate cloning, this original *Bsa*I site will cut itself off and be absent from the ligation product. The other, longer primer must introduce a point mutation within the original *Bsa*I recognition site, and must contain an additional terminal *Bsa*I site that generates the same overhang as the original internal *Bsa*I site, thus making the generated ends ligation-compatible. As an example, this procedure is visualized for an alternative cloning strategy for the construction of pKO-082340 in Supplementary Figure S5d. This strategy is also suitable for coding sequences, because at least two potential wobble positions within the hexameric recognition site of *Bsa*I can be targeted to introduce a translationally silent point mutation without changing the amino acid sequence of the gene product. For further information about this procedure, see Engler et al. (2008).

It should be noted that using the above-described mutation strategy it is also feasible to pre-clone fragments of a GOI or ROI with internal *Bsa*I sites into a Golden Gate vector and then use this vector to amplify the full-length GOI or ROI in one step for subsequent cloning steps.

In conclusion, we generated vector sets for gene fusion and gene deletion in diverse fungi and showed their applicability in two filamentous ascomycetes. Importantly, pDest-Amp can be used as a destination vector for cloning any given fragment(s) that carry the appropriate *Bsa*I sites and is highly useful beyond fungal experimental systems. Ultimately, one can clone complete vectors from various fragments using the Golden Gate strategy, without the need for any destination vector, making the system globally applicable.

## Supplementary Information

Below is the link to the electronic supplementary material.

Supplementary file1 (PDF 782 KB)

## Data Availability

All Golden Gate plasmids generated in this study are available upon request.
