# Supplementary material for: Golden Gate vectors for efficient gene fusion and gene deletion in diverse filamentous fungi
Source: Curr Genet. 2020 Dec 24;67(2):317–30. doi: 10.1007/s00294-020-01143-2 (PMC8032637; doi:10.1007/s00294-020-01143-2)
Supplement: Supplementary file 1 — Supplementary file1 (PDF 782 KB) [file 294_2020_1143_MOESM1_ESM.pdf]

Supplementary material

Golden Gate vectors for efficient gene fusion and gene deletion in diverse filamentous fungi

Current Genetics

Tim Dahlmann, Dominik Terfehr, Kordula Becker, Ines Teichert

Allgemeine und Molekulare Botanik, Ruhr-Universität Bochum, Universitätsstrasse 150, 44801 Bochum, Germany

Corresponding author:
Ines Teichert
ines.teichert@rub.de

Table S1 Fungal strains used in this study

**Strain**	**Description**	**Reference**
P. chrysogenum		
P2niaD18	*niaD*⁻	(Specht et al. 2014)
Δku70FRT2	Δ*ku70*::*FRT*, *niaD*⁻	(Kopke et al. 2010)
ΔEN45-082340	Δ*ku70*::*FRT*, Δ*EN45_082340*::*trpC(p)::nat1*, *niaD*⁻	This study
ΔEN45-100120	Δ*ku70*::*FRT*, Δ*EN45_100120*::*trpC(p)::nat1*, *niaD*⁻	This study
mRFP-MAT1-1-1	*gpdA(p)*::*mRFP*::*MAT1-1-1*, *trpC(p)*::*nat1*, *niaD*⁻	This study
EGFP-MAT1-1-1	*gpdA(p)*::*egfp*::*MAT1-1-1*, *trpC(p)*::*nat1*, *niaD*⁻	This study
S. macrospora		
wt	Wild type; R19027	FGSC #10222
fus	Reddish-brown ascospores; S70823	(Nowrousian et al. 2012)
Δidc3	S143456; Δidc3::*trpC(p)::hph*	This study
Δku70	Δku70::*trpC(p)::nat1*	(Pöggeler and Kück 2006)
Δidc3::G-IDC3 (TKK24A-1)	Δidc3 S143456 transformed with pGGN-G-IDC3; expresses GFP-IDC3; hph^r, nat^r	This study
Δidc3::R-IDC3 (TIT71C-2)	Δidc3 S143456 transformed with pGGN-R-IDC3; TIT71C-2; expresses mRFP-IDC3; hph^r, nat^r	This study
Δidc3::F-IDC3 (TIT2D-2)	Δidc3 S143456 transformed with pGGN-F-IDC3; expresses FLAG-IDC3; hph^r, nat^r	This study
Δidc3::IDC3-G (TKK26C-1)	Δidc3 S143456 transformed with pGGC-G-IDC3; expresses IDC3-GFP; hph^r, nat^r	This study
Δidc3::IDC3-R (TKK27D-1)	Δidc3 S143456 transformed with pGGC-R-IDC3; expresses IDC3-mRFP; hph^r, nat^r	This study
Δidc3::IDC3-F (TIT6B-2)	Δidc3 S143456 transformed with pGGC-F-IDC3; expresses IDC3-FLAG; hph^r, nat^r	This study
TIT26C-1S3	Primary Δtih transformant; Δtih_FLP; Δtih::*trpC(p)::nat::Smxyl(p)::Pcflp*; hph^r, nat^r	This study
IT1513<br>IT1515<br>IT1517<br>IT1535	Ascospore isolates from cross of TIT26C-1S3 to wt Δtih; hph^s, nat^s, brown spores, fertile	This study
TIT8B-1S1<br>TIT8B-1S2<br>TIT8B-1S3	Ascospore isolates<br>Δefd16::*trpC(p)::hph*	This study

hph^r, resistant to hygromycin B; nat^r, resistant to nourseothricin

Table S2 Plasmids used in this study

Plasmid	Description	Reference
pBluescriptIISK(+)	Cloning vector; *bla*	Stratagene
pDest-Amp	Destination vector for Golden Gate cloning; *bla* (Bsamut), *lacZ* gene with two internal *Bsa*I sites *BsaI(4)* and *BsaI(7)*	This study
pD-Phleo	*trpC(p)::phleo*	(Hoff et al. 2010a; Hoff et al. 2010b)
pDrive	PCR cloning vector; *bla, kan*, *lacZ α*	Qiagen
pDrivehph	*trpc(p)::hph* in pDrive; *bla, kan*	(Nowrousian and Cebula 2005)
pEHN1nat	*gpd(p)::egfp::trpC(t); bla, nat1*	(Dreyer et al. 2007)
pEHN2	*gpd(p)::MCS::trpC(t); hph; tel*	(Pöggeler and Kück 2004)
pEHN5	Derivative of pEHN2 without telomeric sequences *gpd(p)::MCS::trpC(t); hph*	This study
pEHN8	Derivative of pEHN5 with new multiple cloning site	This study
pEHN8nat	*gpd(p)::MCS::trpC(t); bla*, *nat1*	This study
pEHN8nat-GG	pEHN8nat without *Bsa*I sites	This study
pFlip	flp/*FRT* vector; *FRT::hph::trpC(p)::smxyl(p)::Pcflp::FRT, bla, kan*	(Bloemendal et al. 2014)
pFlip-mut2	pFlip with two mutated *Bsa*I recognition sites in the *Pcflp* gene	This study
pFlip-mut2_Bsa-Linker1	pFlip-mut2 with one *Bsa*I linker	This study
pGFP-MAT1	*gpd(p)::egfp::Pcmat1-1-1::trpC(t); nat1*	(Becker et al. 2015)
pGGC-F	*gpd(p)::BsaI(1)::lacZ::BsaI(3)::linker::3xflag::trpC(t); bla*, *nat1*	This study
pGGC-F-IDC3	*gpd(p)::idc3::linker::3xflag::trpC(t); bla*, *nat1*	This study
pGGC-G	*gpd(p)::BsaI(1)::lacZ::BsaI(3)::linker::egfp::trpC(t); bla*, *nat1*	This study
pGGC-G-IDC3	*gpd(p)::idc3::linker::egfp::trpC(t); bla*, *nat1*	This study
pGGC-R	*gpd(p)::BsaI(1)::lacZ::BsaI(3)::linker::mRFP::trpC(t); bla*, *nat1*	This study
pGGC-R-IDC3	*gpd(p)::idc3::linker::mRFP::trpC(t); bla*, *nat1*	This study
pGG-Flip-hph	*BsaI(5)::Pcfpl::(Smxyl(p)::trpC(p)::hph::BsaI(6); bla*	This study
pGG-Flip-nat1	*BsaI(5)::Pcfpl::(Smxyl(p)::trpC(p)::nat1::BsaI(6)*; bla	This study
pGG-hph	*BsaI(6)::trpC(p)::hph::BsaI(5)* in pDrive; *bla, kan*	This study
pGG-nat1	*BsaI(5)::trpC(p)::nat1::BsaI(6)* in pJet1.2; *bla*	This study
pGGN-F	*gpd(p)::3xflag::linker::BsaI(1)::lacZ::BsaI(2)::trpC(t); bla*, *nat1*	This study
pGGN-F-IDC3	*gpd(p)::3xflag::linker::idc3::trpC(t); bla*, *nat1*	This study

Plasmid	Description	Reference
pGGN-G	*gpd(p)::egfp::linker::BsaI(1)::lacZ::BsaI(2)::trpC(t); bla*, *nat1*	This study
pGGN-G-IDC3	*gpd(p)::egfp::linker::idc3::trpC(t); bla*, *nat1*	This study
pGGN-G-Pcmat1	*gpd(p)::egfp::linker::Pcmat1-1-1::trpC(t); bla*, *nat1*	This study
pGGN-R	*gpd(p)::mRFP::linker::BsaI(1)::lacZ::BsaI(2)::trpC(t); bla*, *nat1*	This study
pGGN-R-IDC3	*gpd(p)::mRFP::linker::idc3::trpC(t); bla*, *nat1*	This study
pGGN-R-Pcmat1	*gpd(p)::mRFP::linker::Pcmat1-1-1::trpC(t); bla*, *nat1*	This study
pGG-phleo	*BsaI(5)::trpC(p)::phleo::BsaI(6)* in pDrive; *bla, kan*	This study
pIG1783-1	*gpd(p)::egfp::trpC(t); bla, hph*	(Pöggeler et al. 2003)
pJet1.2	PCR cloning vector; *bla*	Thermo Fisher Scientific
pKO-082340	Deletion vector for *P. chrysogenum EN45_082340*	This study
pKO-100120	Deletion vector for *P. chrysogenum EN45_100120*	This study
pKO-efd16	Deletion vector for *S. macrospora efd16*; *bla, hph*	This study
pKO-idc3	Deletion vector for *S. macrospora idc3*; *bla, hph, ura3*	This study
pKO-tih	Deletion vector for *S. macrospora tih*; *bla, nat1*; based on pGG-Flip-nat1 enabling marker recycling	This study
pMSH	*gpd(p)::mRFP1(Sm)Q66T::trpC(t); nat1*<br>*mRFP1* codon-optimized for *S. macrospora* encoding Q66T variant amplified with RFP3 (aaccatggcctcctccgaggacgt) and RFP4 (ttgcggccgcttaggcgccggtggagtg) cloned *Nco*I-*Not*I in pEHN8	(Engh et al. 2010)
pMSHnat	*gpd(p)::mRFP1(Sm)Q66T::trpC(t); nat1*<br>*mRFP1* codon-optimized for *S. macrospora* encoding Q66T variant	This study
pRHN1nat	*gpd*(*p*)::*DsRed*::*trpC*(*t*); *nat1*<br>based on pRHN1 (Janus et al. 2007), but with *nat1* instead of *hph*	Hoff and Kück, unpublished
pRS426	Target vector for yeast recombination; *ura3*, *bla*	(Christianson et al. 1992)
pRS-Kassette	*ura3(p)::URA, smxyl(p)::Pcflp, trpC(p)::hph*	(Bloemendal et al. 2014)
pRS-nat-Kassette	Derivative of pRS-Kassette, containts *nat1* instead of *hph* gene<br>*ura3(p)::URA, smxyl(p)::Pcflp, trpC(p)::nat1*	This study

Table S3 Oligonucleotides used for cloning and verification of deletion strains

**Oligonucleotide**	**Sequence (5'-3')[a]**
082340-3fw	GTACGGTCTCGGTCAGCATTTCAAGTGATGGTCCC
082340-3rv	CTCAGGTCTCCCGTAAACACTGCCGTCACTGGAAC
082340-5fw	GACTGGTCTCAAGTCCAATCTCTATGTCCACACCG
082340-5rv	CAGAGGTCTCAGCAGTCGAAGAGAGACAGTAAGTG
100120-3fw	GTACGGTCTCGGTCAGTTTATCTAGTTTGCGAGCTCC
100120-3rv	CTCAGGTCTCCCGTAGAAAGAGCACCGTCGGCAAC
100120-5fw	GACTGGTCTCAAGTCTTAGGTAAATCGGCCGTTCG
100120-5rv	CAGAGGTCTCAGCAGAGAGGAATATGGTTGTAGGGAC
214-3fw	*gcccaaaaatgctccttcaatatcagttgc*GTACGGGTGCCGCTGTGTGTGGTTG
214-3rv	*gcggataacaatttcacacaggaaacagc***GGATCC**TTACGGGCATTACCTGGGATGCGTG
214-5fw	*gtaacgccagggttttcccagtcacgacg***GGATCC**CTTTCTTCGCCGTCAAGGCCCCGGC
214-5rv	*cgagggcaaaggaatagggttccgttgagg*TTTGGATGTGTGTTGATGTGTTGTG
214-vp1	CTGATGCACTTTGCCCTTGG
214-vp2	CTGCTTACTTCAAGGGCAAG
214-vp3	CTTTCCATACGCTAGATCGG
2986-3fw	CCCATAGGTCTCG*GTCA*TCAAGGTCCAAAGTTATGTCT
2986-3rv	CCCATAGGTCTCG*CGTA*TTTTTTTCAGTCTTTTCTCCTTTTC
2986-5fw	CCCATAGGTCTCC*AGTC*GAGTAATCTTACCAAACTGGC
2986-5rv	CCCATAGGTCTCC*GCAG*GATGAAACTTGTGTGATTTAAACA
2986-vp1	TGCCAGGATTAGCTGTAGTC
2986-vp2	TTTGGAGACCATCTGGGAGG
2986-vp3	TGGCTGTTGGTGATCCTTGG
5650-vp1	CCTCTAGGTATCTCTCAAGG
5650-vp2	CATACATTGAGACGGCAACG
5650-vp3	GAGGGTGTCGATGAGTATGC
BsaI-delete-fw	TTACTTGGCCATCGGTCTCC
BsaI-delete-rv	CCCATAGGTCTCCAGGTGTAGACCGATGGCCAAGTAAATG
Bsa-L1-fw	CGAT**TACGTA**GGTCTCCTGACCTGCAGAACC
Bsa-L1-rv	GGTT**CTGCAG**GTCAGGAGACCTACGTAATCG
Bsa-L2-fw	CCTG**AAGCTT**GCAGTGAGACCAGATCTACCG
Bsa-L2-rv	CGGT**AGATCT**GGTCTCACTGCAAGCTTCAGG
ClaI_PlacZ_f	CTG**ATCGAT**CGACAGGTTTCCCGACTGGA
EGFP_GS_BsaI_ClaI_r	TCG**ATCGAT**GGTCTCG*ACCA*GAGCCAGAACCCTTGTACAGCTCGTCCATGC
FLAG-for1	**CTAGT**GGTCTCA*GGAT*CTGGCTCTGGTATGGACTACAAAGACCATGACGGTGATTATAAAGATCATGATATCGACTACAAGGATGACGATGACAAGTGA**G**

Oligonucleotide	Sequence (5'-3')[a]
FLAG-for3	**CTAGC**ATGGACTACAAAGACCATGACGGTGATTATAAAGATCATGATATCGACTACAAGGATGACGATGACAAGGGTTCTGGCTC*TGGT*CGAGACC**AT**
FLAG-rev1	**GATCC**TCACTTGTCATCGTCATCCTTGTAGTCGATATCATGATCTTTATAATCACCGTCATGGTCTTTGTAGTCCATACCAGAGCCAG*ATCC*TGAGACC**A**
FLAG-rev3	**CGAT**GGTCTCG*ACCA*GAGCCAGAACCCTTGTCATCGTCATCCTTGTAGTCGATATCATGATCTTTATAATCACCGTCATGGTCTTTGTAGTCCAT**G**
GG_Int1	AG**GAATTC**GAAGTCGGAGACCTGACGGTCTCATACGTACGAATTCGC
GG_Int2	GC**GAATTC**GTACGTATGAGACCGTCAGGTCTCCGACTTCGAATTCCT
GG_phleo_fw	GTATGGTCTCACTGCACAACTGATATTGAAGGAGCATTTTTTGG
GG_phleo_rv	CACTGGTCTCCTGACTTCATGAGATGCCTGCAAGCAAT
GG-1	**ACTAGT**GGTCTCA*GGTT*CTGGCTCTGGTATGGTGAGCAAGGGCGAGG
GG-2	**GGATCC**CCGCTTTACTTGTACAGCTCGTCC
GG-3neu	**ATCGAT**C*TGGT*CGAGACCCGACAGGTTTCCCGACTGG
GG-4	**ACTAGT**TTACAATTTCCATTCGCCATTCAGGC
GG-C-mRFP-fw	**ACTAGT**GGTCTCA*GGAT*CTGGCTCTGGTATGGCCTCCTCCGAGGACG
GG-C-mRFP-rv	**GGATCC**CCGCTTTAGGCGCCGGTGGAGTGG
GG-KO-hph-fw	CACTGGTCTCA*CTGC*CCTCAACGGAACCCTATTCC
GG-KO-hph-rv	ACGTGGTCTCA*TGAC*GCAACTGATATTGAAGGAGC
GG-nat1-fw	GTATGGTCTCACTGCACGAATTCAACTGATATTGAAGGAGC
GG-nat1-rv	CACTGGTCTCCTGACTTCAGGGGCAGGGCATGCTC
GG-N-mRFP-fw	**GCTAG**CATGGCCTCCTCCGAGGACG
GG-N-mRFP-rv	**ATCGAT**GGTCTCG*ACCA*GAGCCAGAACCGGCGCCGGTGGAGTGGCG
GGN-PcMAT1-rev	GGGACATGGTCTCC*TACG*CTAGTTGTGCCCAAAGATCC
GG-PcMAT1-for	GGGACATGGTCTCC*TGGT*ATGTCTACCTCTCTTGATGC
hph1MN	CGATGGCTGTGTAGAAGTACTCGC
hph2MN	ATCCGCCTGGACGACTAAACCAA
KO-082340-5fw	TGGTTCTCATACCCGCTCTG
KO-100120-3rw	ACATATCTGGATCGTGACCGG
L-01	**GCGGCCGC**ATAGATCTCAGGTACCTGGAATTCGAGCTAGCTACCGCGGCAATCGATCG**ACTAGT**
L-02	**ACTAGT**CGATCGATTGCCGCGGTAGCTAGCTCGAATTCCAGGTACCTGAGATCTAT**GCGGCCGC**
LacZ_BsaI_SpeI_r	TCG**ACTAGT***TACG*TGAGACCTTACAATTTCCATTCGCC
Mut_pehn8nat_1	CGGTCGTCAAGgGACCTACGAGACTG

**Oligonucleotide**	**Sequence (5’-3’)**[a]
Mut_pehn8nat_2	TTGATCTGCTTGATCTCGTCTCCC
Mut_pehn8nat_3	GGGCGAGCTCTGTACAGT
Mut_pehn8nat_4	GGTTTAGGGTTAGGGCCC
nat1-fw	GGCGCTCTACATGAGCATG
nat-Kass-fw	*tacctattctacccaagcatccaa*ATGGCCACCCTCGACGACAC
nat-Kass-rv	*caactggttcccggtcggcatctact*TCAGGGGCAGGGCATGCTC
NheI_EGFP_f	CTG**GCTAGC**ATGGTGAGCAAGGGCGAGGA
pcflp_mut1_fw	TCGTCACCGAaACCAAGACCT
pcflp_mut1_rv	GGCACTGGATAATGACGC
pcflp_mut2_fw	TCAAGGACGAaACCAATCCCA
pcflp_mut2_rv	GGGCGATCATTTCCTTGG
PtrpC-rw	CTCCACTAGCTCCAGCCAAG
SM#848	TGATACCGCGgGACCCACGC
SM#849	TTGCAGCACTGGGGCCAG
tih-KO-3fw	GGGAAAGGTCTCC*GCAG*GAGAGTGAGTCAGACACTG
tih-KO-3rv	GGGAAAGGTCTCC*CGTA***CATATG**GCTGACAACTTTGGTCTG
tih-KO-5fw	GGGAAAGGTCTCG*AGTC***CATATG**CTGCCAAATTCACATGGG
tih-KO-5rv	GGGAAAGGTCTCG*GTCA*ACCATATACCTGCCGGCT

[a] Restriction sites for cloning are indicated in bold letters, *Bsa*I sites are underlined, *Bsa*I sites with mutations are doubly underlined, *Bsa*I-generated overhangs are indicated in italics, and mutated bases are indicated in lowercase letters. Overlaps for homologous recombination in yeast are shown in lowercase italics. Note that FLAG oligonucleotides were designed such that annealing already provided compatible ends.

Table S4 Golden Gate protocols

		I<br>Standard	II<br>Deletion vectors	III<br>Internal *Bsa*I sites
Initital restriction	37 °C	30 min	90 min	30-90 min, depending on construct
Restriction/ligation	2 min 37 °C<br>+<br>5 min 16 °C	50 cycles	50 cycles	50 cycles
Terminal restriction	37 °C	5 min	5 min	-
	55 °C	5 min	5 min	-
Heat inactivation	80 °C	5 min	5 min	5 min
Storage	11 °C	∞	∞	∞

Table S5 Oligonucleotides for generating and sequencing Golden Gate plasmids as well as generating split marker deletion cassettes. See table S6 for correlation of oligonucleotides and plasmids.

Oligonucleotide	Sequence (5'-3')[a]	Specificity
**Oligonucleotides for amplification of GOIs and flanking regions**		
GG-C/N-fw	CGAGTCAGGTCTCC*TGGT*N$_{[17-23]}$	Forward primer for amplification of GOI for gene fusion<br>*Bsa*I(1) site
GG-N-rv	CGAGTCAGGTCTCC*TACG*N$_{[17-23]}$	Reverse primer for amplification of GOI for N-terminal tagging<br>*Bsa*I(2) site
GG-C-rv	ACCGACAGGTCTCG*ATCC*N$_{[17-23]}$	Reverse primer for amplification of GOI for C-terminal tagging<br>*Bsa*I(3) site
GG-5fw	ACGACTGGTCTCA*AGTC*N$_{[17-23]}$	Forward primer for amplification of 5' flanking region<br>*Bsa*I(4) site
GG-5rv	ATCAGAGGTCTCA*GCAG*N$_{[17-23]}$	Reverse primer for amplification of 5' flanking region<br>*Bsa*I(5) site
GG-3fw	TCGTACGGTCTCG*GTCA*N$_{[17-23]}$	Forward primer for amplification of 3' flanking region<br>*Bsa*I(6) site
GG-3rv	ATCTCAGGTCTCC*CGTA*N$_{[17-23]}$	Reverse primer for amplification of 3' flanking region<br>*Bsa*I(7) site
**Oligonucleotides for sequencing of Golden Gate plasmids**		
1751	GCCATATTTTCCTGCTCTCC	*gpd*(p) forward
1757	AGCTGACATCGACACCAACG	*trpC*(t) reverse
egfp-fw	GGTGAACTTCAAGATCCG	*egfp*, forward
egfp-rv	ACTTGTGGCCGTTTACGTCG	*egfp*, rv reverse
mRFP-fw	CTACAAGACCGACATCAAGC	*mRFP*, forward
mRFP-rv	GAACTCGTGGCCGTTGACGGAGCC	*mRFP*, reverse
426-15	TTGTGTGGAATTGTGAGCGG	pDest-Amp, sequences reverse into 3'-flanking region of deletion vectors
426-14	TTAAGTTGGGTAACGCCAGG	pDest-Amp, sequences forward into 5'-flanking region of deletion vectors
flp-for	TCCCTGACCACTACTTCG	*Pcflp* gene, forward; sequences reverse into 5'-

		flanking region (pGG-Flip-nat1, pGG-Flip-hph)
hph1IT	GGCTGTGTAGAAGTACTCGC	*hph* gene, forward; sequences reverse into 5'-flanking region (pGG-hph) or forward into 3' flanking region (pGG-Flip-hph)
hph2IT	ATCCGCCTGGACGACTAAAC	*trpC*(p), reverse; sequences forward into 3'-flanking region (pGG-hph) or reverse into 5' flanking region (pGG-nat1, pGG-phleo)
nat1-seq	GGCGCTCTACATGAGCATG	*nat1*, forward; sequences forward into 3'-flanking region (pGG-nat1, pGG-Flip-nat1)
phleo-seq	CATCCAGGAAACCAGCAGCG	*phleo*, forward; sequences forward into 3'-flanking region (pGG-phleo)
**Oligonucleotides for split marker PCR**		
hph_split_5´_rv	CGTTGCAAGACCTGCCTGAAACC	*hph* forward; use with GG-5fw to amplify 5' split fragment (if cassette from pGG-hph) or with GG-3rv to amplify 3' split fragment (if cassette from pGG-Flip-hph)
hph_split_3´_fw	TTGGCGACCTCGTATTGGGAATC	*hph* reverse; use with GG-3rv to amplify 3' split fragment (if cassette from pGG-hph) or with GG-5fw to amplify 5' split fragment (if cassette from pGG-Flip-hph)
nat-split-fw_new	TGCGTTGACGTTGGTGAC	*nat1* reverse; use with GG-5fw to amplify 5' split fragment
nat_split_rev_new	CACTGGATGGGTCCTTCAC	*nat1* forward; use with GG-3rv to amplify 3' split fragment

[a] *Bsa*I sites are underlined and *Bsa*I overlaps are indicated in italics.

Table S6 Overview of Golden Gate cloning protocols with vectors from this study

**N-terminal fusion**			
Primers	GG-C/N-fw / GG-N-rv		
Amplified region	GOI, including start and stop codon		
Destination vector	pGGN-F	pGGN-G	pGGN-R
Golden Gate components[a]	PCR, destination vector<br>Equimolar (40-80 fmol each)		
Golden Gate protocol	I		
Sequencing primers	1751, 1757	egfp-fw, 1757	mRFP-fw, 1757
**C-terminal fusion**			
Primers	GG-C/N-fw / GG-C-rv		
Amplified region	GOI, including start codon, excluding stop codon		
Destination vector	pGGC-F	pGGC-G	pGGC-R
Golden Gate components[a]	PCR, destination vector<br>Equimolar ratio (40-80 fmol each)		
Golden Gate protocol	I		
Sequencing primers	1751, 1757	1751, egfp-rv	1751, mRFP-rv
**Deletion vector, standard**			
Primers	GG-5fw / GG-5rv, GG-3fw / GG-3rv		
Amplified region	1 kb each 5'- and 3'-flanking region bordering the genomic region to be deleted		
Destination vector	pDest-Amp		
Cassette donor vector	pGG-hph	pGG-nat1	pGG-phleo
Golden Gate components[a]	5'-PCR, 3'-PCR, destination vector, cassette donor vector<br>Molar ratio 2:2:2:1 (40-80 fmol each)		
Golden Gate protocol	II		
Sequencing primers	hph1IT, hph2IT,<br>426-14, 426-15	nat1-seq, hph2IT,<br>426-14, 426-15	phleo-seq, hph2IT,<br>426-14, 426-15
**Deletion vector for marker recycling**			
Primers	GG-5fw / GG-5rv, GG-3fw / GG-3rv		
Amplified region	1 kb each 5'- and 3'-flanking region bordering the genomic region to be deleted		
Destination vector	pDest-Amp		
Cassette donor vector	pGG-Flip-hph	pGG-Flip-nat1	
Golden Gate components[a]	5'-PCR, 3'-PCR, destination vector, cassette donor vector<br>Molar ratio 2:2:2:1 (40-80 fmol each)		
Golden Gate protocol	II		
Sequencing primers	hph1_IT, flp-for,<br>426-14, 426-15	nat1-seq, flp-for,<br>426-14, 426-15	
**Generation of deletion cassette**			
PCR	GG-5fw / GG-3rv		
Split marker PCR	pGG-hph:<br>GG-5fw / hph_split_5´_rv and hph_split_3´_fw / GG-3rv<br>pGG-Flip-hph:<br>GG-5fw / hph_split_3'-fw and hph_split_5´_rv / GG-3rv<br>pGG-nat1, pGG-Flip-nat1:<br>GG-5fw / nat-split-fw_new and nat_split_rev_new / GG-3rv		
Restriction	*Hind*III and *Pst*I (if no recognition sites in flanking regions)		

[a] All Golden Gate reactions are carried out in a 0.5 ml PCR tube in a total volume of 15 µl in 1x ligation buffer with 1 µl T4 ligase and 1 µl *Bsa*I or *Bsa*IHFv2 (see Materials and Methods).

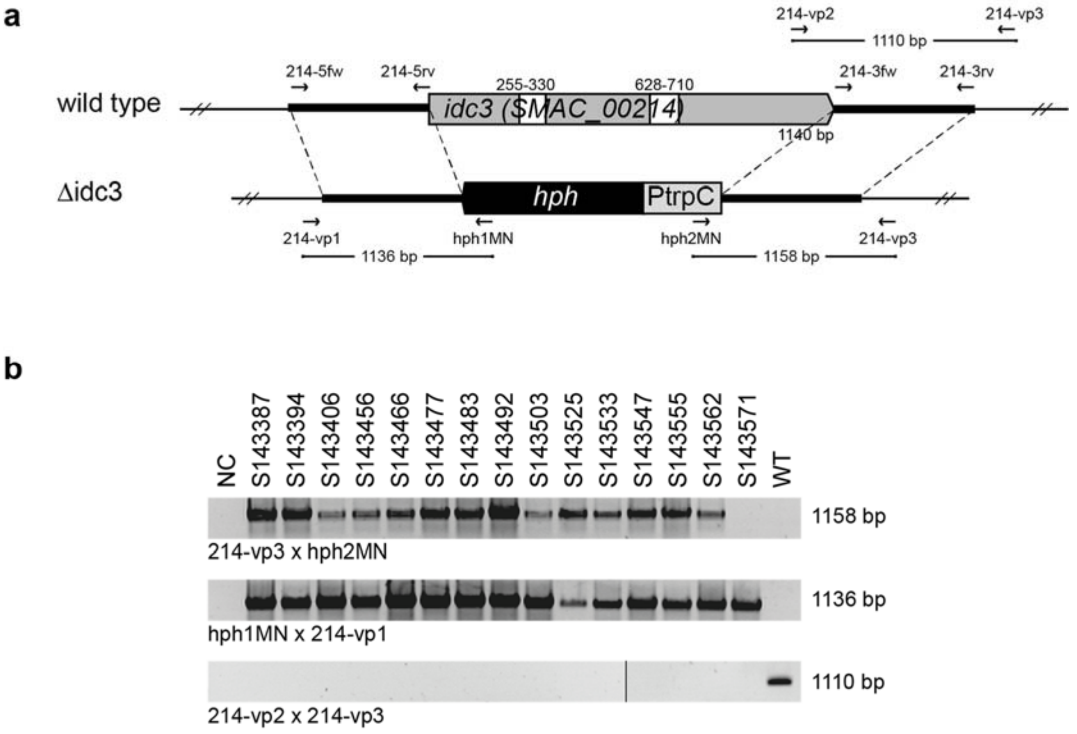

Fig. S1 Generation of *SMAC_00214 (idc3)* deletion strains. **a** Schematic representation of the *idc3* (*SMAC_00214*) genomic locus in wildtype and deletion strains (Δidc3). ORFs are displayed as grey arrows, introns as white boxes. Flanking sequences used for homologous integration of deletion constructs are shown as thick black lines. Black arrows represent primers used for PCR; size of PCR products is given on the thin black lines depicting the PCR products. Not drawn to scale. **b** PCR analysis of Δidc3 ascospore isolates with primers indicated in **a**. Strain S143456 was used in further analysis in this study. NC, negative control

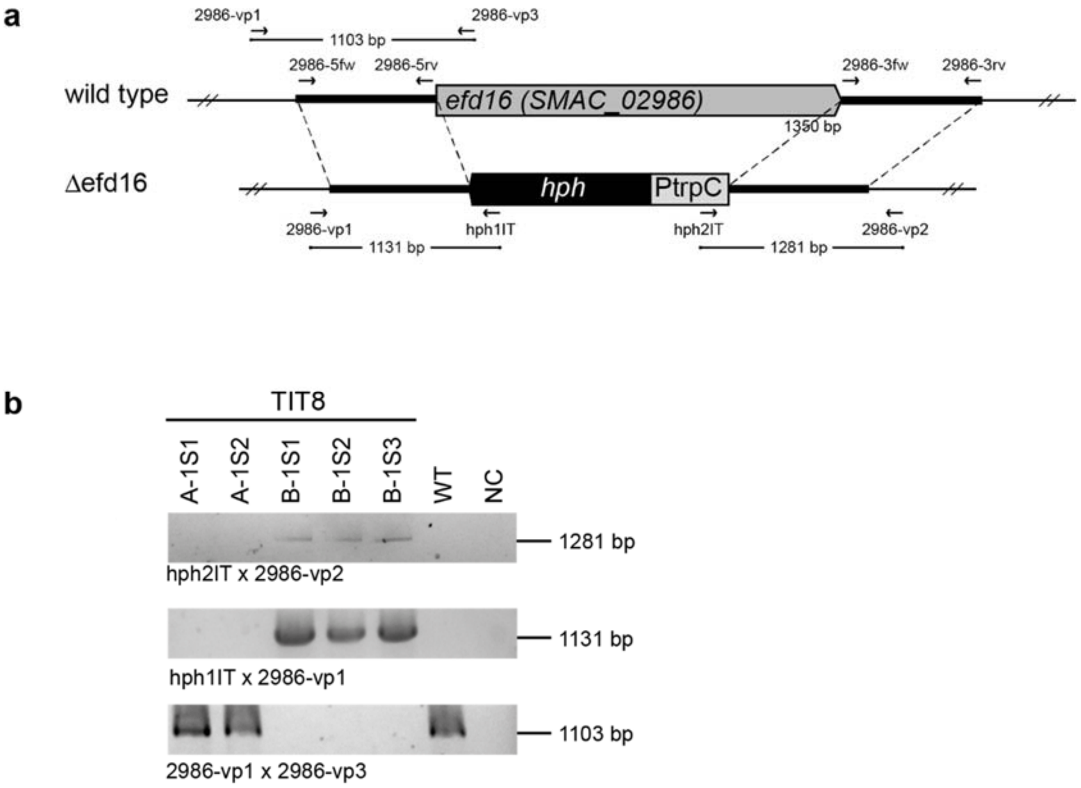

Fig. S2 Generation of *efd16* deletion strains. **a** Schematic representation of the *efd16* (*SMAC_02986*) genomic locus in wildtype and deletion strains (Δefd16). ORFs are displayed as grey arrows. Flanking sequences used for homologous integration of deletion constructs are shown as thick black lines. Black arrows represent primers used for PCR; size of PCR products is given on the thin black lines depicting the PCR products. Not drawn to scale. **b** PCR analysis of putative Δefd16 strains with primers indicated in **a**. Note that TIT8B-1S1, TIT8B-1S2 and TIT8B-1S3 are Δefd16 ascospore isolates. NK, negative control

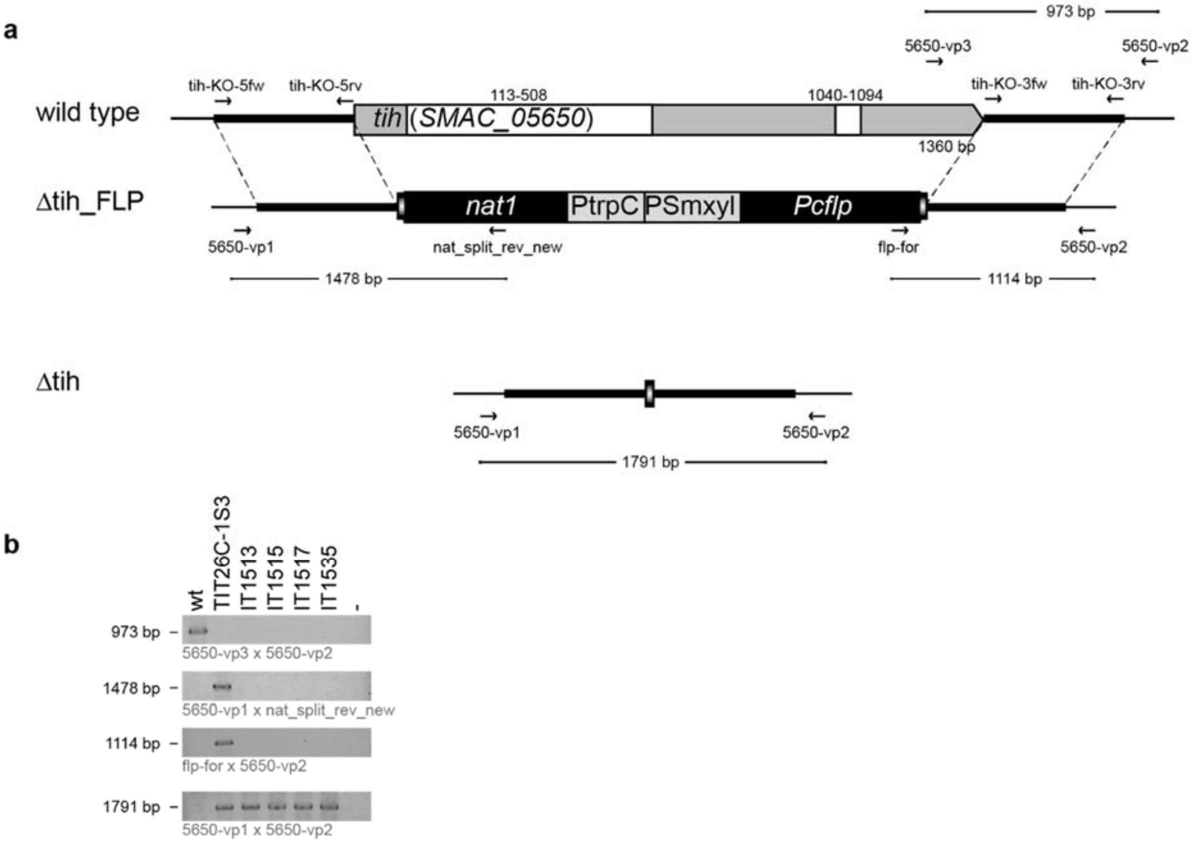

Fig. S3 Generation of *tih* deletion strains. **a** Schematic representation of the *tih* (*SMAC_05650*) genomic locus in wildtype and deletion strains (Δtih_FLP) as well as marker-free Δtih after marker recycling. ORFs are displayed as grey arrows, with introns marked as white boxes. Flanking sequences used for homologous integration of deletion constructs are shown as thick black lines. Black arrows represent primers used for PCR; size of PCR products is given on the thin black lines depicting the PCR products. Not drawn to scale. **b** PCR analysis of Δtih strains with primers indicated in **a**. Note that TIT26C-1S3 is a Δtih_FLP strain still containing the complete deletion cassette, while IT1513, IT1515, IT1517 and IT1535 are Δtih strains derived from crosses of Δtih_FLP to wildtype and contain just one *FRT* site. -, negative control

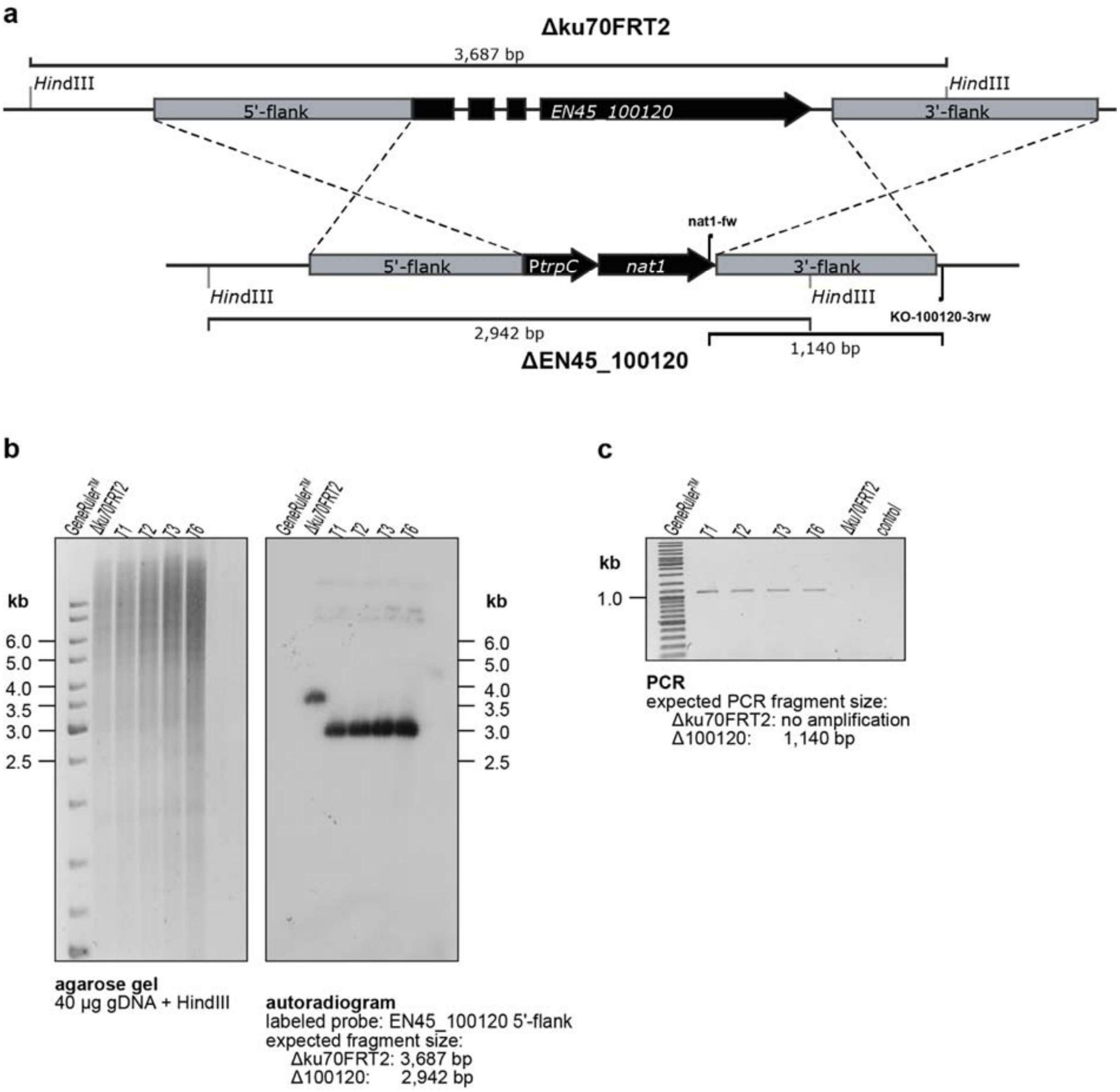

Fig. S4 Generation of *EN45_100120* deletion strains. **a** Schematic representation of the *EN45_100120* genomic locus in the recipient strain Δku70FRT2 and deletion strains (ΔEN45_100120). ORFs are displayed as black arrows. Flanking sequences used for homologous integration of deletion constructs are shown as grey bars. Sizes of PCR products and digestion fragments are given on the thin black lines depicting the DNA fragments, which are drawn to scale. Small black arrows indicate primer binding positions used for PCR. **b** Southern blot analysis of *Hind*III digested genomic DNA of the recipient and ΔEN45_100120 strains with a radioactively labeled probe for the *EN45_100120* 5'-flank. **c** PCR analysis of the correct integration of the deletion cassette at the *EN45_100120* 3'-flank

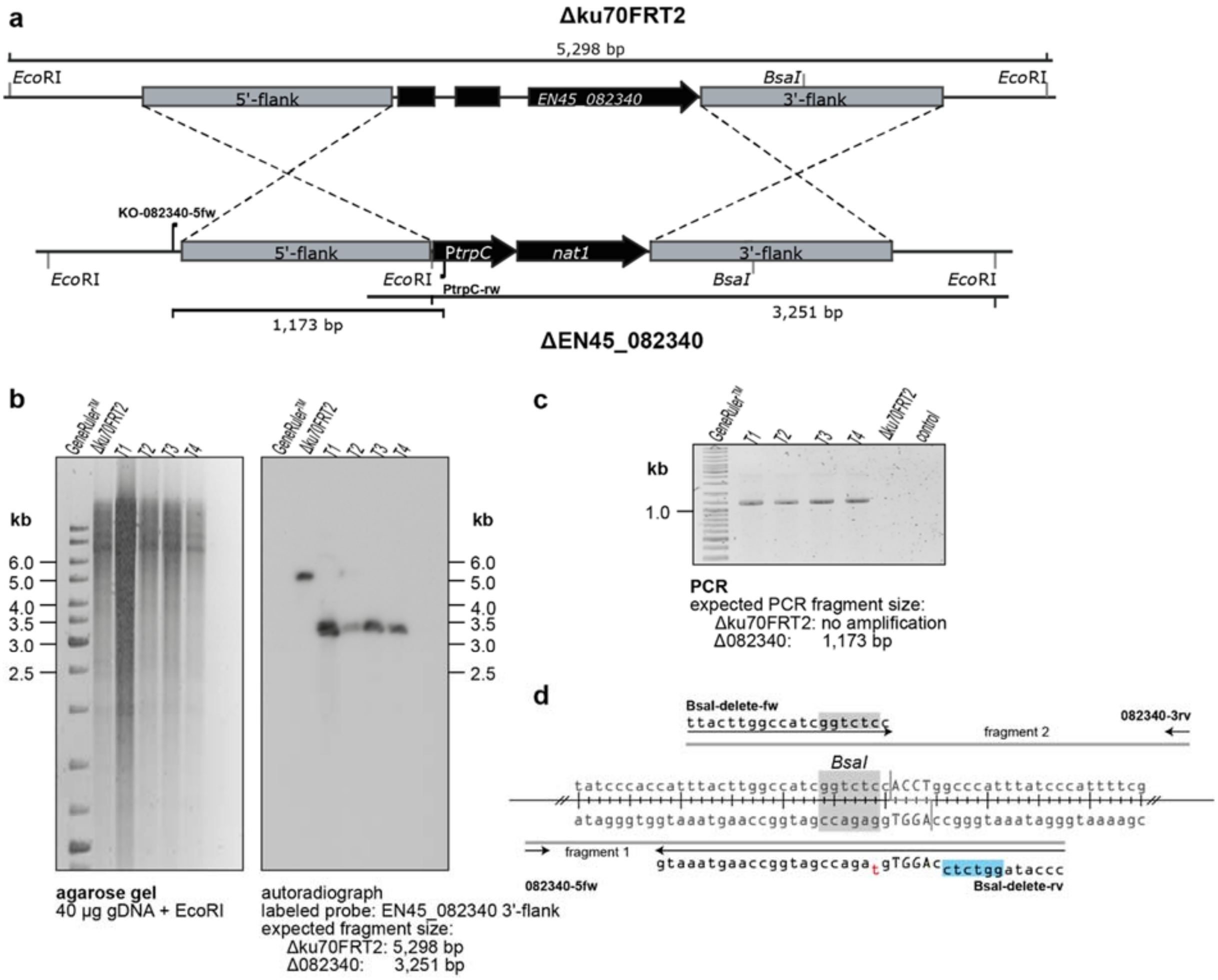

Fig. S5 Generation of *EN45_082340* deletion strains. **a** Schematic representation of the *EN45_082340* genomic locus in the recipient strain Δku70FRT2 and deletion strains (ΔEN45_082340). ORFs are displayed as black arrows. Flanking sequences used for homologous integration of deletion constructs are shown as grey bars. Sizes of PCR products and digestion fragments are given on the thin black lines depicting the DNA fragments, which are drawn to scale. Small black arrows indicate primer binding positions used for PCR. **b** Southern blot analysis of *Eco*RI digested genomic DNA of the recipient and ΔEN45_082340 strains with a radioactively labeled probe for the *EN45_082340* 3'-flank. **c** PCR analysis of the correct integration of the deletion cassette at the *EN45_082340* 5'-flank. **d** Visualization of the primer design necessary for cloning of fragments containing internal *Bsa*I sites. The sequence shows part of the 3'-flank of *EN45_082340* with the internal *Bsa*I binding site *ggtctc* (grey box, facing right and generating a 4 nt overhang (capital letters). The short primer BsaI-delete-fw contains the original *Bsa*I site (grey box) that will cut itself off from the PCR fragment generated with primer pair BsaI-delete-fw / 082340-3rv (fragment 2). Thus, this fragment will no longer contain the original internal *Bsa*I binding site. The longer primer BsaI-delete-rv will introduce a point mutation (red) into the original *Bsa*I binding site. For generation of an overhang that is ligation-compatible to the overhang in fragment 2, another *Bsa*I site has to be introduced into the primer (blue box). This *Bsa*I site will also cut itself off from the PCR fragment generated with primer pair 082340-5fw / BsaI-delete-rv (fragment 1). Both, the original *Bsa*I site as well as the one added for cloning (blue box) will be absent from the ligation product after successful Golden Gate cloning.

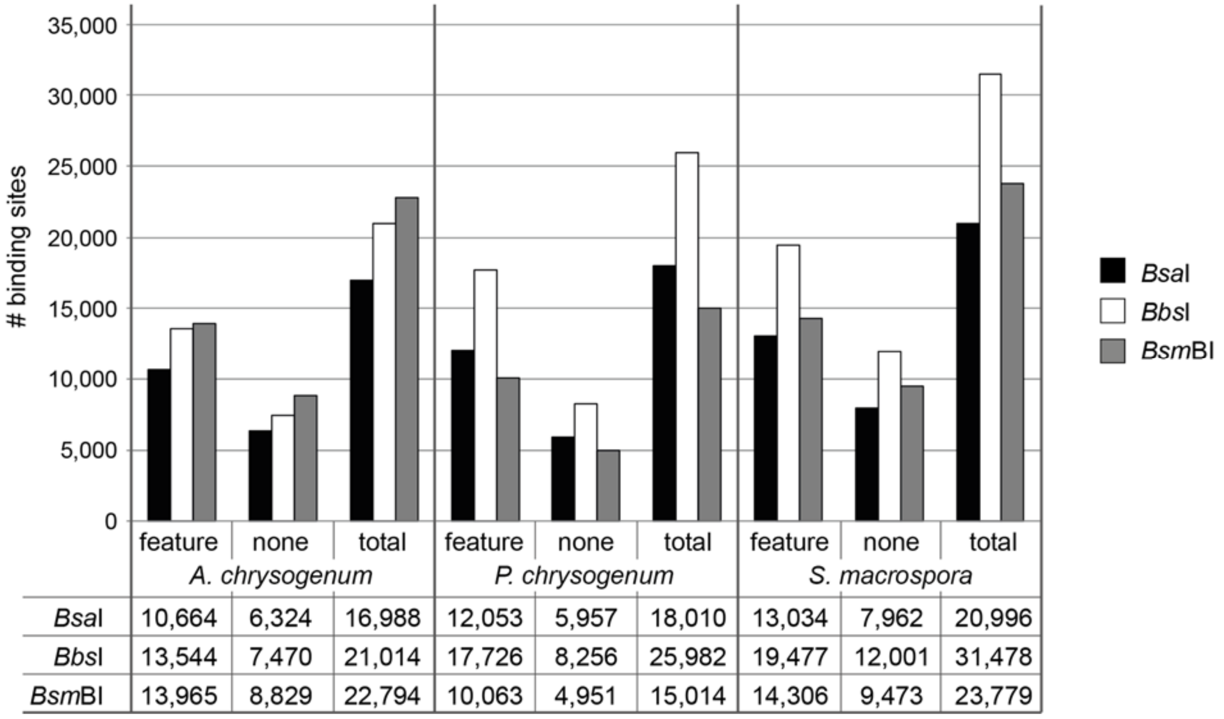

	A. chrysogenum			P. chrysogenum			S. macrospora		
	feature	none	total	feature	none	total	feature	none	total
*Bsa*I	10,664	6,324	16,988	12,053	5,957	18,010	13,034	7,962	20,996
*Bbs*I	13,544	7,470	21,014	17,726	8,256	25,982	19,477	12,001	31,478
*Bsm*BI	13,965	8,829	22,794	10,063	4,951	15,014	14,306	9,473	23,779

Fig. S6 Distribution of type IIS restriction sites in fungal genomes. Binding sites for type IIS restriction endonucleases *Bsa*I (black), *Bbs*I (white) and *Bsm*BI (grey) in annotated features (feature), regions without annotated features (none) as well as total sites for *Bsa*I, *Bbs*I, and *Bsm*BI are given for each genome. The recognition sites were counted within the genomes of *A. chrysogenum* ATCC 11550, *P. chrysogenum* P2niaD18, and *S. macrospora* k-hell FGSC 10222 by custom-made Perl scripts (Teichert et al. 2012; Specht et al. 2014; Terfehr et al. 2014)

References

Becker K, Beer C, Freitag M, Kück U (2015) Genome-wide identification of target genes of a mating-type alpha-domain transcription factor reveals functions beyond sexual development. Mol Microbiol 96:1002-1022

Bloemendal S, Löper D, Terfehr D, Kopke K, Kluge J, Teichert I, Kück U (2014) Tools for advanced and targeted genetic manipulation of the beta-lactam antibiotic producer *Acremonium chrysogenum*. J Biotechnol 169:51-62

Christianson TW, Sikorski RS, Dante M, Shero JH, Hieter P (1992) Multifunctional yeast high-copy-number shuttle vectors. Gene 110:119-122

Dreyer J, Eichhorn H, Friedlin E, Kürnsteiner H, Kück U (2007) A homologue of the *Aspergillus velvet* gene regulates both cephalosporin C biosynthesis and hyphal fragmentation in *Acremonium chrysogenum*. Appl Environ Microbiol 73:3412-3422

Engh I, Nowrousian M, Kück U (2010) *Sordaria macrospora*, a model organism to study fungal cellular development. Eur J Cell Biol 89:864-872

Hoff B, Kamerewerd J, Sigl C, Mitterbauer R, Zadra I, Kürnsteiner H, Kück U (2010a) Two components of a velvet-like complex control hyphal morphogenesis, conidiophore development, and penicillin biosynthesis in *Penicillium chrysogenum*. Eukaryot Cell 9:1236-1250

Hoff B, Kamerewerd J, Sigl C, Zadra I, Kück U (2010b) Homologous recombination in the antibiotic producer *Penicillium chrysogenum*: strain ΔPcku70 shows up-regulation of genes from the HOG pathway. Applied Microbiology and Biotechnology 85:1081-1094

Janus D, Hoff B, Hofmann E, Kück U (2007) An efficient fungal RNA-silencing system using the *DsRed* reporter gene. Appl Environ Microbiol 73:962-970

Kopke K, Hoff B, Kück U (2010) Application of the *Saccharomyces cerevisiae* FLP/FRT recombination system in filamentous fungi for marker recycling and construction of knockout strains devoid of heterologous genes. Appl Environ Microbiol 76:4664-4674

Nowrousian M, Cebula P (2005) The gene for a lectin-like protein is transcriptionally activated during sexual development, but is not essential for fruiting body formation in the filamentous fungus *Sordaria macrospora*. BMC Microbiol 5:64

Nowrousian M, Teichert I, Masloff S, Kück U (2012) Whole-Genome Sequencing of Sordaria macrospora Mutants Identifies Developmental Genes. G3 (Bethesda) 2:261-270

Pöggeler S, Kück U (2004) A WD40 repeat protein regulates fungal cell differentiation and can be replaced functionally by the mammalian homologue striatin. Eukaryotic Cell 3:232-240

Pöggeler S, Kück U (2006) Highly efficient generation of signal transduction knockout mutants using a fungal strain deficient in the mammalian *ku70* ortholog. Gene 378:1-10

Pöggeler S, Masloff S, Hoff B, Mayrhofer S, Kück U (2003) Versatile EGFP reporter plasmids for cellular localization of recombinant gene products in filamentous fungi. Current Genetics 43:54-61

Specht T, Dahlmann TA, Zadra I, Kürnsteiner H, Kück U (2014) Complete Sequencing and Chromosome-Scale Genome Assembly of the Industrial Progenitor Strain P2niaD18 from the Penicillin Producer *Penicillium chrysogenum*. Genome Announc 2

Teichert I, Wolff G, Kück U, Nowrousian M (2012) Combining laser microdissection and RNA-seq to chart the transcriptional landscape of fungal development. BMC Genomics 13:511

Terfehr D, Dahlmann TA, Specht T, Zadra I, Kürnsteiner H, Kück U (2014) Genome sequence and annotation of *Acremonium chrysogenum*, producer of the β-lactam antibiotic cephalosporin C. Genome Announc 2:e00948-00914
